# Structural Brain Imaging Phenotypes of Mild Cognitive Impairment (MCI) and Alzheimer's Disease (AD) Found by Hierarchical Clustering

**DOI:** 10.1155/2020/2142854

**Published:** 2020-11-13

**Authors:** Mikko Kärkkäinen, Mithilesh Prakash, Marzieh Zare, Jussi Tohka, for the Alzheimer's Disease Neuroimaging Initiative

**Affiliations:** ^1^A.I. Virtanen Institute for Molecular Sciences, University of Eastern Finland, Kuopio, Finland; ^2^Signal Processing, Tampere University, Tampere, Finland; ^3^Alzheimer's Disease Neuroimaging Initiative Study, USA

## Abstract

A hierarchical clustering algorithm was applied to magnetic resonance images (MRI) of a cohort of 751 subjects having a mild cognitive impairment (MCI), 282 subjects having received Alzheimer's disease (AD) diagnosis, and 428 normal controls (NC). MRIs were preprocessed to gray matter density maps and registered to a stereotactic space. By first rendering the gray matter density maps comparable by regressing out age, gender, and years of education, and then performing the hierarchical clustering, we found clusters displaying structural features of typical AD, cortically-driven atypical AD, limbic-predominant AD, and early-onset AD (EOAD). Among these clusters, EOAD subjects displayed marked cortical gray matter atrophy and atrophy of the precuneus. Furthermore, EOAD subjects had the highest progression rates as measured with ADAS slopes during the longitudinal follow-up of 36 months. Striking heterogeneities in brain atrophy patterns were observed with MCI subjects. We found clusters of stable MCI, clusters of diffuse brain atrophy with fast progression, and MCI subjects displaying similar atrophy patterns as the typical or atypical AD subjects. Bidirectional differences in structural phenotypes were found with MCI subjects involving the anterior cerebellum and the frontal cortex. The diversity of the MCI subjects suggests that the structural phenotypes of MCI subjects would deserve a more detailed investigation with a significantly larger cohort. Our results demonstrate that the hierarchical agglomerative clustering method is an efficient tool in dividing a cohort of subjects with gray matter atrophy into coherent clusters manifesting different structural phenotypes.

## 1. Introduction

Alzheimer's disease (AD) is the most common neurodegenerative disease and cause of dementia [1]. The characteristic early symptoms of Alzheimer's disease are short-term memory loss, language problems, disorientation, mood swings, and behavioral issues. The shrinkage of the cerebral cortex and the medial temporal lobe is a typical trait of Alzheimer's disease along with the enlargement of brain ventricles [2]. The extracellular amyloid plaques and intraneuronal tangles of hyperphosphorylated tau protein have been widely recognized as central markers of Alzheimer's disease [3, 4].

Genetic variation and different environmental exposures lead to heterogeneities in neurodegenerative patterns. Finding and classifying these patterns (clusters) using sophisticated computer-aided tools and thereby grouping the subjects to more homogeneous groups can be clinically useful [1, 5–7]. In particular, it would be beneficial to be able to predict the onset of AD by applying computational tools to examine the MRIs. Towards this end, data clustering methods from applied mathematics have found increasing applications in neuroscience [8]. The goal of these methods is to group or cluster the subjects by maximizing a certain similarity condition, which is typically a numerical metric that can be calculated for two clusters. Subjects falling into the same cluster may have similarities in the pathogenesis of MCI and AD which may elucidate the disease mechanisms especially when genetic, demographic, and clinical data are incorporated. Several clustering algorithms exist [8]: connectivity-based clustering or hierarchical clustering, centroid-based clustering, and distribution-based or density-based clustering. For higher-dimensional data, more recent developments such as CLIQUE have gained some popularity [9].

Clustering methods appear well suited to the task of dividing the subjects into different categories based on structural phenotypes as manifested by various disease subtypes at different stages of disease progression. Previous works in this field have found different structural phenotypes of MCI/AD subjects with computational methods, including clustering methods, as summarized in Table 1 [5–7, 10–18]. For example, of the three AD subtypes investigated in [16, 17], the hippocampal-sparing subtype of AD (i.e., cortically driven atrophy and relative sparing of the hippocampus) showed more aggressive progression (as measured by the cognitive MMSE and ADAS ratings) than the typical AD and the limbic-predominant AD. The typical AD and the limbic-predominant AD were found by Ferreira et al. to have the worst clinical progression rate of CDR (Clinical Dementia Rating) and MMSE (Mini-Mental State Examination) decline, while the hippocampal-sparing and no atrophy subtypes showed less aggressive progression [11]. The varying rates of decline are thought to be driven by cortical atrophy that is worst in younger hippocampal-sparing AD subtype, while the limbic-predominant subtype shows more severe hippocampal atrophy and less cortical atrophy [14, 16, 17]. The typical AD (or late-onset AD, LOAD) manifests both atrophy patterns quite equally [16]. Results obtained by clustering can be interpreted bearing in mind the potential of the clustering algorithms to identify different subtypes of MCI/AD pathology. Patients with the hippocampal-sparing subtype of AD died younger and a higher proportion of them were men, as observed in, e.g., [16]. Those with limbic-predominant AD are typically older and a higher proportion of them are women. The neurofibrillary tangle count which is strongly related to amnesia is higher in the hippocampus with the limbic-predominant subtype than with the hippocampal-sparing subtype [10]. The APOE *ε*4 is thought to play a slightly smaller role in the hippocampal-sparing atypical subtype of AD than in other subtypes [16].

The main focus of this work is in quantifying the differences between the various emerging structural phenotypes found with the hierarchical clustering methods and discussing the phenotypes in light of existing knowledge. Our results verify that agglomerative hierarchical clustering can be used for classifying patterns of gray matter atrophy, and our results are aligned with existing results for AD subjects obtained with different methods. For MCI subjects, we observe more diverse patterns of atrophy calling for further investigation of the pathogenesis and structural changes related to MCI.

Out of the publications based on clustering algorithms in Table 1 [5, 7, 12], many have the limitation of needing to set the number of clusters based on a priori information. Instead, in hierarchical clustering, applied in this paper, the number of clusters (subtypes) can be decided based on data. Differently from other hierarchical clustering algorithms [5, 12], our agglomerative distance-based clustering method considering a cohort including MCI, AD, and control subjects is able to produce a large number of clusters at one stroke manifesting different structural phenotypes. As will be subsequently discussed, the agreement of our results with previously published and acknowledged results obtained with entirely different methods is generally good, and we also obtain new structural phenotypes of undetermined significance, interesting material for further research. Furthermore, we present a novel way of analysing clustering applied to the subtype identification based on brain imaging phenotype by illustrating the differences between clusters using statistical parametric mapping.

## 2. Material and Methods

### 2.1. ADNI Data

The ADNI initiative was launched in 2003 as a public & private partnership, led by Principal Investigator Michael W. Weiner, MD. The primary goal of ADNI has been to test whether serial MRI, PET, other biological markers, and clinical and neuropsychological assessment can be combined to measure the progression of MCI and early AD.

ADNI material considered in this work includes all subjects from ADNI1, ADNI-GO, and ADNI2 for whom the baseline MRI data (T1-weighted MP-RAGE sequence at 1.5 Tesla or 3.0 Tesla), typically 256 × 256 × 170 voxels with the voxel size of approximately 1mm × 1mm × 1.2mm was available. This led to a database of 1560 subjects, 1461 of which had a baseline diagnosis, age, APOE, and initial ADAS data available. These 1461 subjects form the sample for this work.

### 2.2. Subjects

The subject characteristics are listed in Table 2. A total of 428 subjects were normal controls (NC) in our cohort, while 751 were diagnosed as suffering from MCI and 282 had Alzheimer's disease (AD) at baseline, see Table 2. There were 805 males and 656 females in our cohort. We used demographic data (sex, age, education in years), APOE *ε*4 genotype, and follow-up data (diagnosis and ADAS score) 0, 12, 24, and 36 months from baseline as clinical auxiliary information. The ADAS scores were used in monitoring disease progression after baseline, and the clinical diagnosis was used to track status changes. The APOE *ε*4 prevalence (at least one allele) in NC subjects was 27.3%, in MCI subjects it is 49.3% and in AD subjects it is 66.7%.

### 2.3. MRI Preprocessing

Preprocessing is essential to render the image data between individual subjects comparable. The preprocessing of the MRI data was done by the fully automated CAT12 pipeline (CAT = Computational Anatomy Toolbox, http://www.neuro.uni-jena.de/cat/). These images are quantitative (each voxel intensity corresponds to the amount of gray matter (GM) in that voxel) and they can be compared voxel-by-voxel thanks to the spatial normalization. The details of image preprocessing can be found in the Appendix. The images resulting from preprocessing are called GM density images.

We removed the confounds by a linear regression technique similar to the one introduced in [19]: having voxel-wise GM density value as the dependent variable, we fitted a linear regression model with age, gender, scanner field strength (binary coded as 1.5T = 0 and 3T = 1), and years of education as independent variables on a voxel-by-voxel basis using the data from NC subjects. Then, this regression model was applied to the data of MCI and AD subjects, and residuals from the model were taken as the variables of interest.

To use hierarchical clustering, we must define a distance between any pair of two images. This imaging phenotypic distance was computed as the Manhattan distance of the voxel intensities over the brain mask. This resulted in a symmetric matrix of distances between all subjects that served as the input for the hierarchical clustering algorithm. We note that all subjects were included into clustering although our main interest lies in MCI and AD subjects.

### 2.4. Clustering Method

We clustered the subjects using the agglomerative hierarchical clustering algorithm with the farthest neighbor metric described in [20], i.e., the complete linkage algorithm. The computation starts with 1461 separate clusters that are progressively merged as the calculations proceed. Every iteration reduces the number of clusters by one by fusing two clusters. The two fused clusters, A and B, are those which have the smallest maximum distance of elements. That is, we find clusters A and B for which *d* = max | *a* − *b*∣ is minimized, where *a* ∈ *A* and *b* ∈ *B*. The choice of clustering method and its parameters are discussed in the Appendix. The clustering method was implemented in Matlab R2018b.

The clustering methodology directly utilized the preprocessed MRI tissue maps while characteristics of the subjects listed in the previous section were used only to as demographic and clinical side information when interpreting the clusters, i.e., no other information than MRI enter to the clustering algorithm. The mean value or average diagnosis is calculated for each cluster as a weighted average of the clinical status (i.e., NC = 1, MCI = 2, AD = 3) within each cluster to help guide attention and to interpret the results. The clusters were divided into three categories based on the weighted average diagnoses.

### 2.5. Cluster Characteristics

Linear regression for the ADAS trajectory of each subject in the cluster was performed to compute the rate of change in the ADAS score and the mean slope (the unit is ADAS points/month), and standard deviation was calculated for each cluster.

### 2.6. Analysing the Differences between Structural Phenotypes

Our main interest is in comparing the GM maps in the clusters found during hierarchical clustering. However, differences in MRIs are barely discernible on visual inspection without special tools. We adopted a standard voxel-based morphometry approach that is widely utilized (e.g., in [21]), whereby the neuroanatomical differences between any two groups can be conveniently compared by voxel-wise *t*-tests on gray matter density images. The *t*-test value (*t*) itself is taken as the parameter to be visualized in each voxel, thus producing a 3D t-map of cluster differences.

## 3. Results

### 3.1. General Characteristics of Clustering

**Figure 1:**
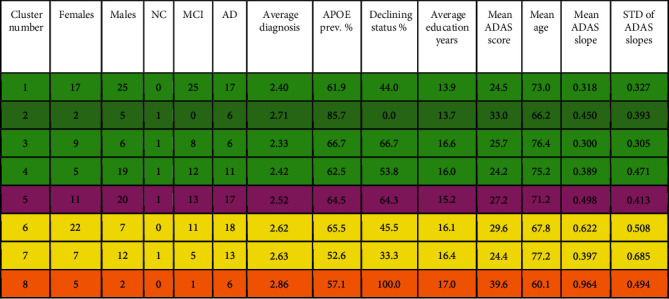
AD clusters and their characteristics. The average diagnosis was calculated as a weighted average of the clinical status (i.e., NC = 1, MCI = 2, and AD = 3). The worsening clinical status column is relevant only from NC to MCI and from MCI to AD progression because the worsening status is deduced from categorical variables. Italic in the Table emphasizes the structural similarity of the cluster phenotypes.

**Figure 2:**
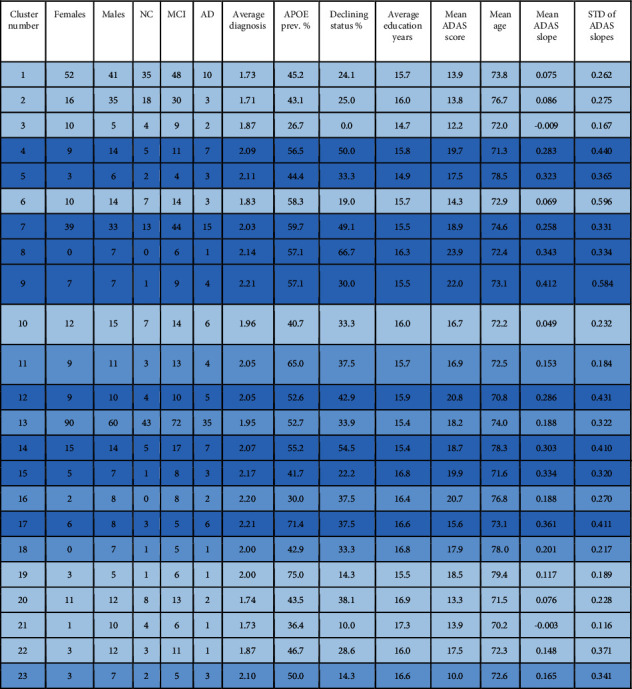
MCI clusters and their characteristics. Italic background color refers to faster progression. The number of MCI clusters is higher than the number of MCI subtypes discussed in many other works because some of our clusters turn out to be structurally very similar.

A total of 8 AD clusters and 23 MCI clusters with interesting characteristics were found. The cluster characteristics are listed in Figures 1 and 2, respectively. Very small clusters (less than 7 subjects) are excluded from the discussion because they were either judged to be outliers or did not allow statistically meaningful analysis. The clusters were categorized as AD, MCI, or normal clusters by considering the average diagnosis. The average diagnosis was calculated as a weighted average of the clinical status (i.e., NC = 1, MCI = 2, and AD = 3). The cluster category was decided simply by dividing the interval from 1 to 3 into 3 equally wide subintervals, i.e., a cluster was an MCI cluster when the average diagnosis was between 1.667 and 2.333; NC cluster if the average diagnosis was at most 1.666; and AD cluster when the average diagnosis was greater than 2.333. This categorization is possible as the distribution of numerically coded diagnoses within clusters never was bimodal, i.e., there were no clusters characterized by the absence of MCI subjects and containing both NC and AD subjects.

The mean ADAS slopes were used to guide our attention amidst the vast number of resulting clusters. The 95% confidence interval for the distribution of mean ADAS slopes for AD clusters (in Figure 1) was [0.309 0.672]; for MCI clusters (in Figure 2), it was [0.138 0.245]; and for NC clusters (in Table 3), it was [-0.027 0.022]. The confidence intervals do not overlap, i.e., when the clusters are organized into these three categories based on weighted average diagnosis, the progression rates of cluster ADAS scores were statistically significantly different between the groups.

### 3.2. Clusters with High Presence of AD Subjects

**Figure 3:**
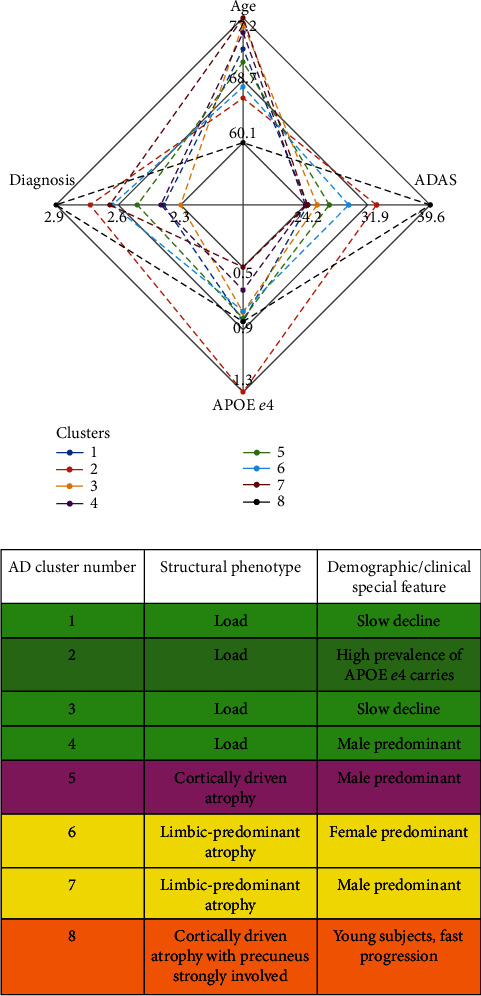
Top panel: radar plot of AD clusters. Cluster 8 consisted of, on average, strikingly young subjects, and the baseline ADAS score was the highest for all clusters. The role of APOE *ε*4 was markedly elevated for cluster 2 as compared with other clusters. Bottom panel: table listing structural phenotypes for AD clusters with demographic and/or clinical features. Clusters 1 and 3 displayed the slowest average decline rate among the AD clusters in terms of the change in ADAS scores during the three-year follow-up. Color coding refers to structural similarity. The same color coding is used in subsequent, more detailed Figure 1.

The characteristics of the AD clusters are illustrated by displaying the key parameters as a radar plot in Figure 3 along with cluster phenotypes and demographic characteristics. More detailed cluster characteristics are listed in Figure 1. Figure 1 includes all the clusters for which the average diagnosis was at least 7/3 and therefore characterized as AD clusters. The high proportion of APOE *ε*4 in these clusters as compared with the rest of the clusters stands out. The cluster-wise mean ADAS slopes were on average higher than with the MCI and NC clusters as already mentioned.

**Figure 4:**
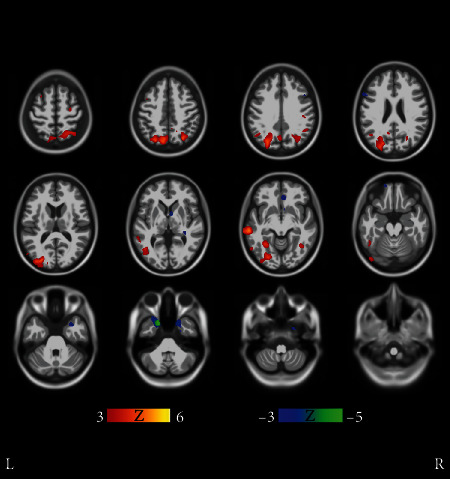
Results of voxel-wise *t*-tests of gray matter distributions between clusters 1 and 2 in Figure 1. The FDR corresponding to ∣*t* | >3 threshold was 0.143. Average *t*-values were not high and differences between the clusters occurred in a scattered manner suggesting that the clusters 1 and 2 manifested similar (typical) AD pathogenesis. Further results (not shown) revealed that clusters 1-4 show a lot of similarity and most likely manifest the typical AD pathogenesis.

We concluded that the atrophy patterns of clusters 1-4 in Figure 1 fit with the typical AD. This conclusion was arrived at for two reasons: First, clusters 1-4 appeared very similar in MRI comparisons, with only small differences visible in MRIs that appeared randomly distributed as seen in more detail in Figure 4. The cluster 2 in Figure 1 was paid special attention because of the quite low average age of 66.2 years and the high baseline ADAS score 33.0 as compared with clusters 1, 3, and 4. Perhaps surprisingly, however, the MRIs did not reveal striking differences in regional atrophy when comparing cluster 2 with clusters 1, 3, and 4. For these reasons, subjects of clusters 1-4 were deemed to most likely follow a similar course of the disease. Secondly, in MRI comparisons of the union of clusters 1-4 manifest marked atrophy as compared with the union of all NC clusters (predominantly healthy controls). Results of this fundamental comparison are shown in Supplementary Results (Figures 11-13), and they fit the neurodegenerative patterns of typical AD where the medial temporal lobe is strongly involved [2].

**Figure 5:**
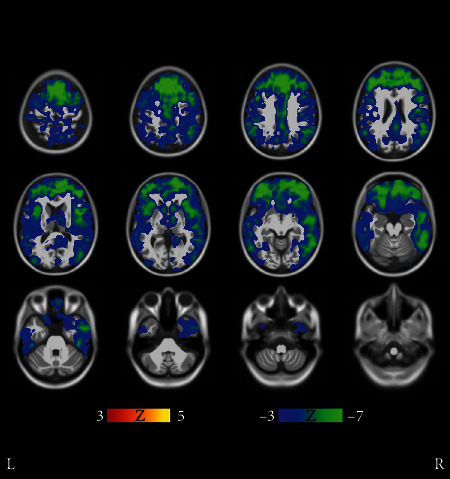
Results of voxel-wise *t*-tests of gray matter distributions between cluster 5 and union of clusters 1-4 in Figure 1. Cluster 5 displayed more cortical atrophy than the union of clusters 1-4, especially in the frontal and temporal lobes. Moreover, the differences were strikingly unidirectional in all brain regions displaying marked differences. Coronal and sagittal views of the same figure are shown in Supplementary Results (Figures 5 b and 5 c). The FDR value corresponding to the threshold ∣*t* | >3 was 0.00135.

**Figure 6:**
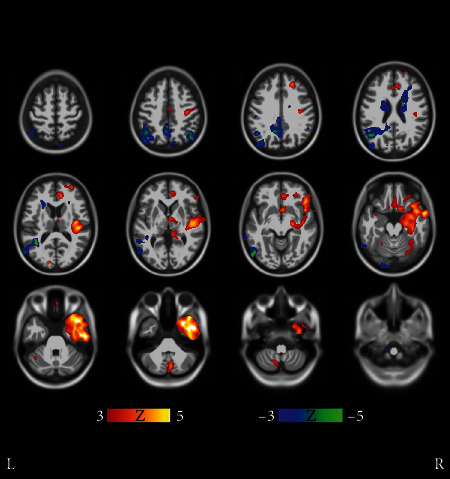
Results of voxel-wise t-tests of gray matter distributions between clusters 6 and 7 in Figure 1. Clusters 6 and 7 very closely resemble each other. Voxel-wise *t*-tests do not show significant regional differences in atrophy patterns of clusters 6 and 7 except retroorbitally on the right hemisphere, where cluster 7 subjects display more atrophy. Due to scattered and anatomically confined bidirectional differences, the clusters are interpreted to consist mostly of subjects having a similar atypical subtype of AD. Coronal and sagittal views are shown in Supplementary Results (Figures 4 b and 4 c). The FDR value is 0.0345.

**Figure 7:**
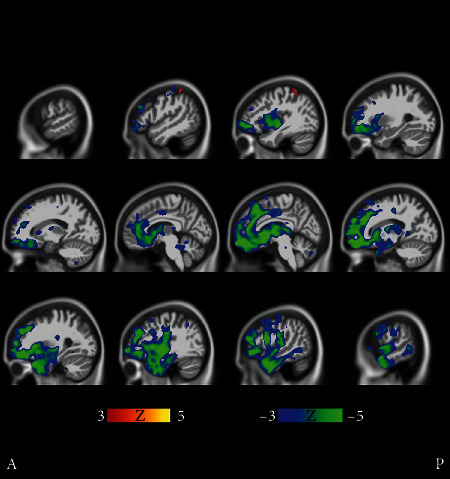
Results of voxel-wise *t*-tests of gray matter distributions between clusters 5 and 6 in Figure 1. Clusters 5 and 6 differ in terms of cortical atrophy (most notably in the frontal lobe) that is more prominent in cluster 5. Coronal and transverse views are shown in Supplementary Results (Figures 5 b and 5 c). The FDR value is 0.0119.

**Figure 8:**
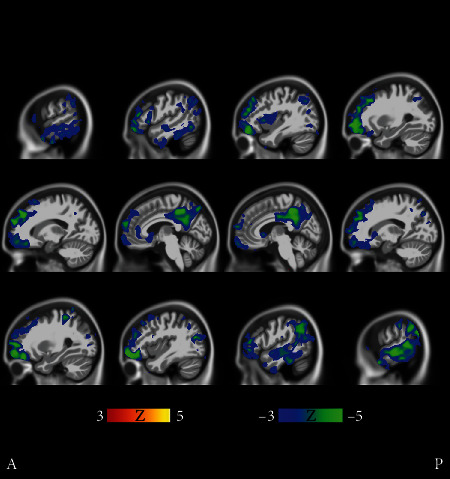
Results of voxel-wise *t*-tests of gray matter distributions between the cluster 8 and the union of clusters 6 and 7 in Figure 1. The precuneus and the frontal lobe of cluster 8 subjects are on average more atrophic than with subjects in clusters 6 and 7. Coronal and transverse views are shown in Supplementary Results (Figures 6 b and 6 c). The FDR value is 0.0109.

Structural atrophy becomes more evident when looking at clusters 5-8, some of them having higher baseline ADAS scores and/or steeper ADAS slopes. Let us take a look at cluster 5 first. Voxel-wise comparisons in Figure 5 reveal remarkable structural differences between cluster 5 and clusters 1-4. Subjects in cluster 5 have, on average, more cortical atrophy in the frontal and temporal lobes than subjects in clusters 1-4. This suggests that the subjects in cluster 5 featured a neurodegenerative pattern deviating from the typical course of the disease, i.e., an atypical AD subtype. While the differences of cortical atrophy are statistically significant in comparison with clusters 1-4 (typical AD), the differences in the hippocampal region are more uncertain. Considering the male preponderance, the aggressive progression, and the more cortically driven atrophy of cluster 5, it seems likely that this cluster would represent an atypical AD subtype where cortical atrophy dominates, with relative sparing of the hippocampi. Clusters 5-8 and the related Figures 5–8 are discussed in more detail in Section 4.

Some subjects with a typical AD subtype are buried in the MCI-dominated clusters. Hence, the role of the atypical subtypes of AD may appear exaggerated, although, in reality, they represent a minor proportion of AD cases. Some of the clusters are mostly formed well before the iteration is terminated, indicating that groups of similar subjects deviating strikingly from the others (compatible with finding atypical MRIs from the cohort) are effectively captured by the algorithm.

### 3.3. Clusters Interpreted as Predominantly MCI

The clusters consisting of predominantly MCI subjects (average diagnosis between 1.67 and 2.33) and their characteristics are shown in Figure 2above. It is immediately clear that these clusters also include many control subjects and some AD subjects. The APOE *ε*4 alleles were less abundant in these clusters than in the AD clusters. The mean ADAS baseline scores and slopes were generally clearly lower than in the AD clusters as was noted in Section 3.1. We quantified the progression rates of the AD, MCI, and NC clusters and noted that they are different, as was pointed out in Section 3.1. We found that the MCI progression rates were distributed between the NC and AD progression rates.

We run the voxel-wise *t*-tests for the MCI clusters in Figure 2 in the same way as with the AD clusters in Figure 1. However, this time, we made no attempt to compare all the clusters as the number of pairwise comparisons increases quadratically as a function of the number of clusters to be compared, 23 clusters resulting in 276 cluster comparisons. Instead, we make some comparisons of the clusters deemed interesting based on cluster characteristics of Figure 2. We note that many of the clusters turned out to be similar. For instance, clusters 16 and 18-23 showed only small differences in brain atrophy patterns. We focus on comparisons between clusters of slow (or stable MCIs) and fast progressors. Clusters 3 and 21 in Figure 2 were selected to represent particularly slow progressors based on their ADAS slopes and small conversion rates. We considered clusters 14, 15, and 17 as a reference of fast progressors because they had the highest ADAS slopes.

**Figure 9:**
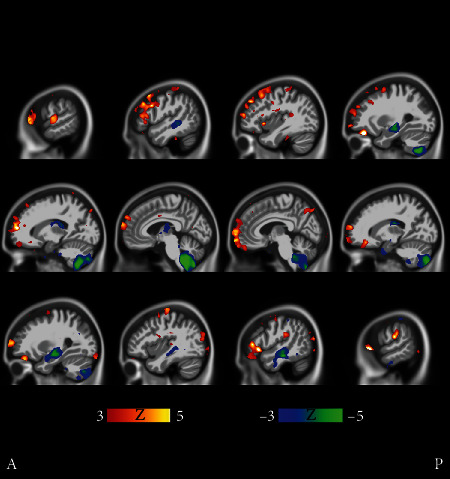
Fast MCI progressors (cluster 9 in Figure 2) compared with stable MCI subjects (cluster 21 in Figure 2). The *t*-tests manifest bidirectional differences. The faster MCI progressors show more atrophy in the medial temporal lobe and especially in the cerebellum, while the stable MCI subjects manifest more atrophy in the frontal cortex, albeit in a scattered manner. Coronal and transverse views are shown in supplementary results (Figures 8 b and 8 c). The FDR value is 0.0510.

Figure 9 illustrates the comparison between the slowest progressing cluster 3 and fast progressors (union of the clusters 14, 15, and 17). The differences in the level of atrophy were striking. Gray matter loss in clusters 14, 15, and 17 appeared nearly in the entire intracranial volume (excluding the occipital lobe and perhaps part of the parietal cortex) as compared with the cluster 3.

Clusters 9 and 21 in Figure 2 show very different progression rates as measured with ADAS slopes. To quantify the differences, the fastest progressing cluster 9 was compared with the stable MCI subjects in cluster 21. The comparison in Figure 9 reveals cortical differences in atrophy, especially in the frontal lobe. These clusters' differences are interesting as the comparison revealed differences in both directions across the brain.

**Figure 10:**
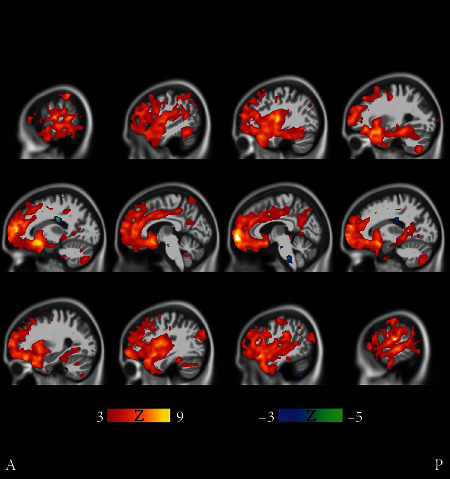
Cluster 8 shows on average significantly more cortical and also subcortical atrophy than cluster 9. All cluster 8 subjects are males, and the baseline ADAS score was the highest of all MCI clusters. Yet, the ADAS slope is highest, i.e., the longitudinal decline of cluster 9 is the worst of all MCI clusters. Transverse and coronal views are shown in Supplementary Results (Figures 9 b and 9 c). The FDR value is 0.00786.

There were regional differences between the fast progressing MCI clusters as can be seen in Figure 10, where clusters 8 and 9 are compared. Cluster 8 was chosen because it has the highest baseline ADAS score of all MCI clusters, close to those of AD clusters in Figure 1. Cluster 9 was the fastest progressing MCI cluster as measured with ADAS slopes, and the baseline ADAS score was the second highest of MCI clusters. In Figure 10, frontocortical and subcortical structures show differences in atrophy, sparking a hypothesis that the MCI of cluster 8 is due to emerging AD. The strikingly unidirectional differences in atrophy suggest that the pertaining etiologies might remarkably deviate from each other.

### 3.4. Clusters Interpreted as Predominantly Normal Controls

The clusters with the lowest average diagnoses are presented in Table 3. The mean ADAS slopes were close to zero as expected. Unsurprisingly, there were many MCI subjects present in these clusters, but only very few AD subjects. As a whole, no striking features with respect to disease progression are evident in Table 3 as expected, and the results are shown here for the sake of completeness.

## 4. Discussion

### 4.1. Methodology

We applied a hierarchical clustering algorithm to MRIs of a cohort of 1461 subjects including AD patients, MCI subjects, and healthy controls. The clustering algorithm was based on a voxel by voxel distances between gray densities of the MRIs normalized to stereotactic space. Therefore, the cluster analysis did not *a priori* target any particular brain region. It can be considered a strength of the method that it considers the whole intracranial volume and is not tuned to find anything particular, yet it produced a multitude of results that are compatible with results obtained with entirely different approaches [5, 12, 14, 16]. Our methodology, as demonstrated, appears particularly useful in searching for entirely new patterns of brain atrophy thus paving the way for finding new (sub)types of AD/MCI pathogenesis.

Because the cerebral cortex contains vastly more voxels than the hippocampus, it appears possible that our method is more sensitive to cortical atrophy than hippocampal atrophy. Therefore, the regions of interest approach with more balanced weights of different regions might be worth exploring to better capture the changes localized in smaller neuroanatomical regions. Hence, an obvious modification to this method would be to restrict to certain neuroanatomical regions hypothesized to be related to different pathologies. This approach might result in even more coherent clusters. Also, a longitudinal investigation of brain atrophy could be done using clustering techniques with comparisons to clinical findings.

### 4.2. Discussion on AD Clusters

The clustering method found 8 AD clusters with more than 7 subjects. The clusters 1-4 (in Figure 1) were deemed typical AD clusters while clusters 5-8 may represent atypical phenotypes that we consider as more interesting. Regarding the structural phenotypes and progression rates of atypical AD subtypes, our results align with [12, 14, 16], particularly with respect to the clusters with cortically driven atrophy patterns (clusters 5 and 8 in Figure 1). On the other hand, the lack of strong cortical atrophy and female preponderance of the subjects in AD cluster 6 in Figure 1 led us to hypothesize that this cluster represents the limbic predominant subtype of AD. In this cluster, the MCI progression rate into AD was remarkable and could indicate that the MCIs would progress into a similar subtype of AD as the diagnosed AD cases in this cluster. Based on a voxel-based analysis of MRIs, cluster 7 resembled very closely cluster 6 (Figure 6). The more prominent cortical atrophy differentiated cluster 5 from clusters 6 and 7 as shown in Figure 7. The demographic and clinical features of clusters 6 and 7 would be consistent with the limbic-predominant subtype of AD. Still, to label the overall pathology of these clusters as limbic-predominant is admittedly rather speculative.

Cluster 8 featured the highest mean baseline ADAS score 39.6 of all clusters and the lowest average age 60.1 years of all clusters at baseline. A structural feature that distinguished the cluster 8 from the other clusters was the marked atrophy near the precuneus as shown in Figure 8. Based on Figure 8, it is evident that the precuneus, known to be involved in episodic memory and visuospatial processing, of the subjects in cluster 8 was more atrophic than that of other clusters. This is an interesting finding because disproportionate atrophy in precuneus has been previously associated with earlier onset of AD and posterior cortical atrophy shows a female bias [21, 22]. Comparing with characteristics presented in [14, 16], we are most likely facing with an atypical AD subtype that is driven by cortical atrophy with parietal cortical atrophy also especially evident. The low age at baseline diagnosis (6 of the 7 subjects were under 60 years old at baseline) and the fast progression rate as calculated from ADAS scores along with the MRI differences in precuneus as compared with other clusters would support this hypothesis. The proportion of cortically driven (or relatively hippocampal-sparing) subtype has been found to be higher than other subtypes in early-onset AD (EOAD) [16]. In Figure 8, statistically significant differences were seen also near the basal ganglia. Volumes of subcortical structures, including the amygdala, hippocampus, thalamus, putamen, globus pallidus, and nucleus caudatus, are known to decrease in AD, showing different rates of decline depending on age [23]. An FDG PET-study showed that the glucose metabolism in the left precuneus of EOAD subjects was markedly impaired as compared with late-onset AD (LOAD) subjects [24]. Interestingly, notable atrophy in the precuneus was the feature that distinguished our AD cluster 8, with low average age at baseline, from the other clusters (see Figure 1 and Figure 8). Additionally, bilateral posterior parietal, posterior cingulate, posterior temporal, and precuneal regions were found to be more vulnerable to atrophy in younger AD subjects [18]. The higher atrophy rate for younger patients that we found was also observed in [18].

### 4.3. Discussion on MCI Clusters

**Figure 11:**
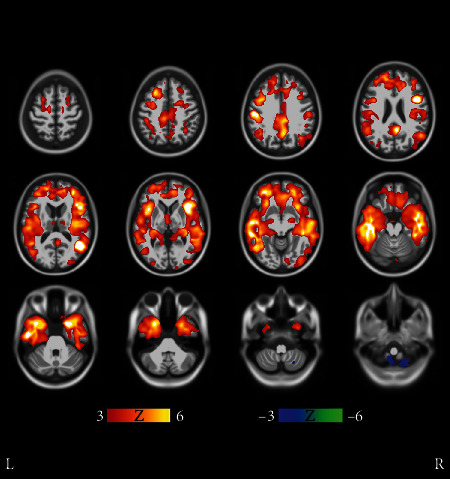
Results of voxel-wise *t*-tests of gray matter distributions between MCI cluster 3 and the union of MCI clusters 14, 15, and 17. The slowest progressing MCI cluster 3 had statistically significantly higher gray matter density than the fastest progressing MCI clusters 14, 15, and 17. Coronal and sagittal views are shown in Supplementary Results (Figures 7 b and 7 c). The FDR value is 0.00673.

**Figure 12:**
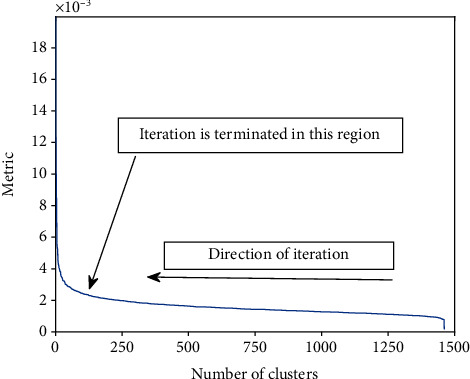
The metric based on which the clusters are merged as a function of the number of clusters. The metric appears rather flat until the number of clusters has decreased to about 200. Within the range of about 50-200 clusters, the internal coherency of the clusters gradually starts degenerating because of “forced” mergers with outliers. Computation proceeds in the direction of decreasing the number of clusters as indicated.

Our MCI results suggest that the structural MCI phenotypes are very diverse and worth exploring in more detail. We were able to recognize clusters of predominantly MCI subjects with different progression rates into AD and varying patterns of brain atrophy (see Figures 9–11). The varying progression rates manifest themselves as a wide array of values from close to zero (stable MCI) to values near those of AD. We found striking heterogeneities in MCI atrophy patterns in the cerebral cortex, subcortical structures, and the anterior part of the cerebellum, where Purkinje cells have been shown to display morphometric changes in AD [25]. Interestingly, the fastest declining (in terms of ADAS slopes) cluster 9 in Figure 2 displayed notable cerebellar atrophy as compared with slower decliners (see Figure 9). This agrees with the previous literature as those MCI subjects that converted to AD were shown to manifest greater cerebellar atrophy than cognitively normal subjects in [26]. Considering our MCI results, it might be tempting to think that subjects in the faster declining MCI clusters might later turn into fast progressing atypical ADs. However, if that were the case, one would anticipate some clusters of strikingly young MCI subjects. But, in fact, none of the MCI clusters have an average age below 70 years. Most likely, many of the MCI subjects progressing fast will simply enter the typical AD subtype. We hypothesize that many of the MCI subjects that will turn into atypical AD did enter the atypical AD clusters in Figure 1 as discussed above rather than the MCI clusters in Figure 2. The number of MCI phenotypes detected by our clustering algorithm was higher than in other works [5, 21]. However, many of these methods are supplied with a predefined number of clusters as opposed to our data-driven number of clusters selection. As MCI is a complex and heterogeneous clinical construct, the number of true MCI subtypes remains a challenging question.

Regarding AD and frontotemporal degeneration (FTD), the behavioral variant of FTD (bvFTD) was differentiated from AD based on gray matter content in the nucleus caudatus and inferior frontal lobe adjacent to the longitudinal fissure (gyrus rectus) in [27]. Interestingly, there is a possibility that some of the MCI subjects in our cohort may be of the frontotemporal type (FT-MCI, [28]). Were that the case, the etiology would most likely be unrelated to APOE *ε*4. Differentiating FTD from MCI/AD is beyond the scope of this work but remains an interesting possibility within the framework of our methodology.

## Figures and Tables

**Table 1:** Methods and key findings of cited literature.

Reference	MCI or AD	Method	Application
[5]	MCI	Multilayer clustering	Identification of rapid and slow decliners
[11]	AD	Visual rating scales	Recognizing AD subtypes
[12]	AD	Random forest pairwise similarity and hierarchical clustering	Varying rates of degeneration of AD subtypes
[7]	AD	*k*-means clustering and support vector machines	Subtypes of AD atrophy
[18]	MCI and AD	Voxel-wise statistical analysis and regression models	Brain atrophy w.r.t age and APOE genotype
[14]	AD	Voxel-based morphometry, statistical analysis using ANOVA	Regional atrophy patterns and progression rates of AD subtypes
[16]	AD	Neurofibrillary tangle count using digital microscopy, statistical methods (ANOVA, *t*-tests)	Subtypes of AD and distinct clinical characteristics
[17]	AD	Cortical, hippocampal volume measurements, statistical methods	Progression rates of AD subtypes
[21]	MCI and AD	Voxel-based morphometry	Atrophy pattern related to progression from MCI to AD
[19]	MCI and AD	Semisupervised machine learning and random forest classification	Predicting conversion from MCI to AD
[22]	AD	Voxel-based morphometry and regression analysis	Precuneus atrophy in early-onset Alzheimer's disease

**Table 2:** APOE *ε*4 data and baseline diagnoses of the subjects. The baseline diagnosis depends on the number of APOE *ε*4alleles (chi-squared test *p* < 0.001).

	Mean ADAS score at baseline	ADAS score std at baseline	No APOE *ε*4	APOE *ε*4 heterozygotes	APOE *ε*4 homozygotes	Σ
NC	9.4	4.3	311	106	11	428
MCI	16.6	6.8	381	290	80	751
AD	29.8	8.0	94	130	58	282
*Σ*	—	—	786	526	149	1461

**Table 3:** The clusters with the lowest average diagnosis (NC = 1, MCI = 2, AD = 3).

Cluster number	Females	Males	NC	MCI	AD	Average diagnosis	APOE prev. %	Declining status %	Mean education years	Mean ADAS score	Mean age	Mean ADAS slope	STD of ADAS slopes
1	22	18	18	22	0	1.55	35.0	27.5	16.1	11.6	74.3	-0.049	0.189
2	17	19	17	15	4	1.64	41.7	34.4	16.1	14.4	73.8	0.057	0.183
3	93	66	71	80	8	1.60	37.7	23.2	16.4	12.6	73.6	0.062	0.434
4	38	39	36	34	7	1.62	31.2	11.4	16.0	12.0	74.7	0.016	0.156
5	16	31	30	17	0	1.36	36.2	17.0	16.1	11.5	76.6	0.017	0.172
6	5	20	10	15	0	1.60	32.0	20.0	15.8	13.3	70.9	-0.040	0.246
7	8	8	8	7	1	1.56	37.5	13.3	17.8	10.8	73.7	0.011	0.179
8	1	7	4	4	0	1.50	25.0	25.0	16.8	9.1	71.8	-0.037	0.142
9	7	6	8	5	0	1.38	15.4	15.4	16.8	10.8	72.3	-0.020	0.156
10	5	17	9	12	1	1.64	31.8	19.0	16.6	13.9	75.1	0.012	0.143
11	3	4	4	3	0	1.43	14.3	14.3	16.0	13.7	80.1	-0.014	0.167
12	3	8	9	2	0	1.18	9.1	9.1	17.0	10.8	71.8	-0.042	0.133

## Data Availability

Data used in preparation of this article were obtained from the Alzheimer's Disease Neuroimaging Initiative (ADNI) database http://adni.loni.usc.edu/.

## Appendix

### A. Methodological Details

#### A1. Image Preprocessing

The CAT12 pipeline first denoised the images using adaptive nonlocal means filtering [29], segmented the images into gray matter (GM), white matter (WM), and cerebrospinal fluid (CSF) [30], computed partial volume fractions [31], and spatially normalized the tissue fraction images (nonaffinely) to the stereotactic MNI space using the DARTEL algorithm [32]. This resulted in spatially aligned GM and WM tissue fraction maps. Thereafter, the tissue fraction maps were smoothed with a Gaussian filter with 8 mm FWHM (full width at half maximum) isotropic kernel. We considered only the GM images as these will include the most salient information for the dementia applications.

#### A2. Choice of Clustering Method and Its Parameters

Regarding the choice of algorithm, we noted that there are some individual outliers, i.e., MRIs that are of poorer quality or for some other reason deviate quite notably from others so that the corresponding subjects do not cluster early during the iteration. The agglomerative clustering is well suited for this setting because the most obvious outliers will be automatically clustered in the later phases of the computation. We look for the clusters in an explorative way by judiciously terminating the iteration before encountering the worse image data thus avoiding an unnecessary dilution of the results.

The standard way to observe the clustering dynamics is to keep track of the clustering metrics as a function of the number of clusters and to consider the so-called elbow plot (Figure 12) in estimating the reasonable number of clusters [20]. The farthest neighbor metric was used in distance-based clustering. Other metrics exist and could be used but after experimenting with other metrics, the farthest neighbor metric was deemed the most appropriate for our purposes because it produced the most coherent clusters.

#### A3.Thresholding of Statistical Maps

For uniform visualization, we decided to threshold the *t*-maps at an uncorrected threshold ∣*t* | >3 and approximate the false discovery rate (FDR) at ∣*t* | = 3. The FDR was approximated by computing *q*-values for each voxel and selecting the *q*-value of the voxel with the minimum ∣*t*∣ larger than 3 as the FDR of the thresholded map [33]. These FDR values, which alert about multiplicity issues, are given in figure captions. We note that as the number of possible cluster comparisons is large; it is necessary to limit the discussion to most illustrative comparisons.

## Supplementary Materials

Supplementary Materials Figure 3(b): coronal view of the image shown in Figure 3. Figure 3(c): sagittal view of the image shown in Figure 3. Figure 4(b): coronal view of the image shown in Figure 4. Figure 4(c): sagittal view of the image shown in Figure 4. Figure 5(b): coronal view of the image shown in Figure 5. Figure 5(c): transverse view of the image shown in Figure 5. Figure 6(b): coronal view of the image shown in Figure 6. Figure 6(c): transverse view of the image shown in Figure 6. Figure 7(b): coronal view of the MRIs in Figure 7. Figure 7(c): sagittal view of the MRIs in Figure 7. Figure 8(b): coronal view of the MRIs in Figure 8. Figure 8(c): transverse view of the MRIs in Figure 8. Figure 9(b): transverse view of the MRIs in Figure 9. Figure 9(c): coronal view of the MRIs in Figure 9. Figure 11: clusters 1-4 from Figure 1 (typical AD) are compared with normal controls (NC). Transverse view, FDR value is 0.000502. Figure 12: clusters 1-4 from Figure 1 (typical AD) are compared with normal controls (NC). Coronal view, FDR value is 0.000502. Figure 13: clusters 1-4 from Figure 1 (typical AD) are compared with normal controls (NC). Sagittal view, FDR value is 0.000502.
