# Supplementary material for: Structural Brain Imaging Phenotypes of Mild Cognitive Impairment (MCI) and Alzheimer's Disease (AD) Found by Hierarchical Clustering
Source: Int J Alzheimers Dis. 2020 Nov 13;2020:2142854. doi: 10.1155/2020/2142854 (PMC7708019; doi:10.1155/2020/2142854)
Supplement: Supplementary Materials — Figure 3(b): coronal view of the image shown in Figure 3. Figure 3(c): sagittal view of the image shown in Figure 3. Figure 4(b): coronal view of the image shown in Figure 4. Figure 4(c): sagittal view of the image shown in Figure 4. Figure 5(b): coronal view of the image shown in Figure 5. Figure 5(c): transverse view of the image shown in Figure 5. Figure 6(b): coronal view of the image shown in Figure 6. Figure 6(c): transverse view of the image shown in Figure 6. Figure 7(b): coronal view of the MRIs in Figure 7. Figure 7(c): sagittal view of the MRIs in Figure 7. Figure 8(b): coronal view of the MRIs in Figure 8. Figure 8(c): transverse view of the MRIs in Figure 8. Figure 9(b): transverse view of the MRIs in Figure 9. Figure 9(c): coronal view of the MRIs in Figure 9. Figure 11: clusters 1-4 from Figure 1 (typical AD) are compared with normal controls (NC). Transverse view, FDR value is 0.000502. Figure 12: clusters 1-4 from Figure 1 (typical AD) are compared with normal controls (NC). Coronal view, FDR value is 0.000502. Figure 13: clusters 1-4 from Figure 1 (typical AD) are compared with normal controls (NC). Sagittal view, FDR value is 0.000502. [file 2142854.f1.pdf]

Supplementary results

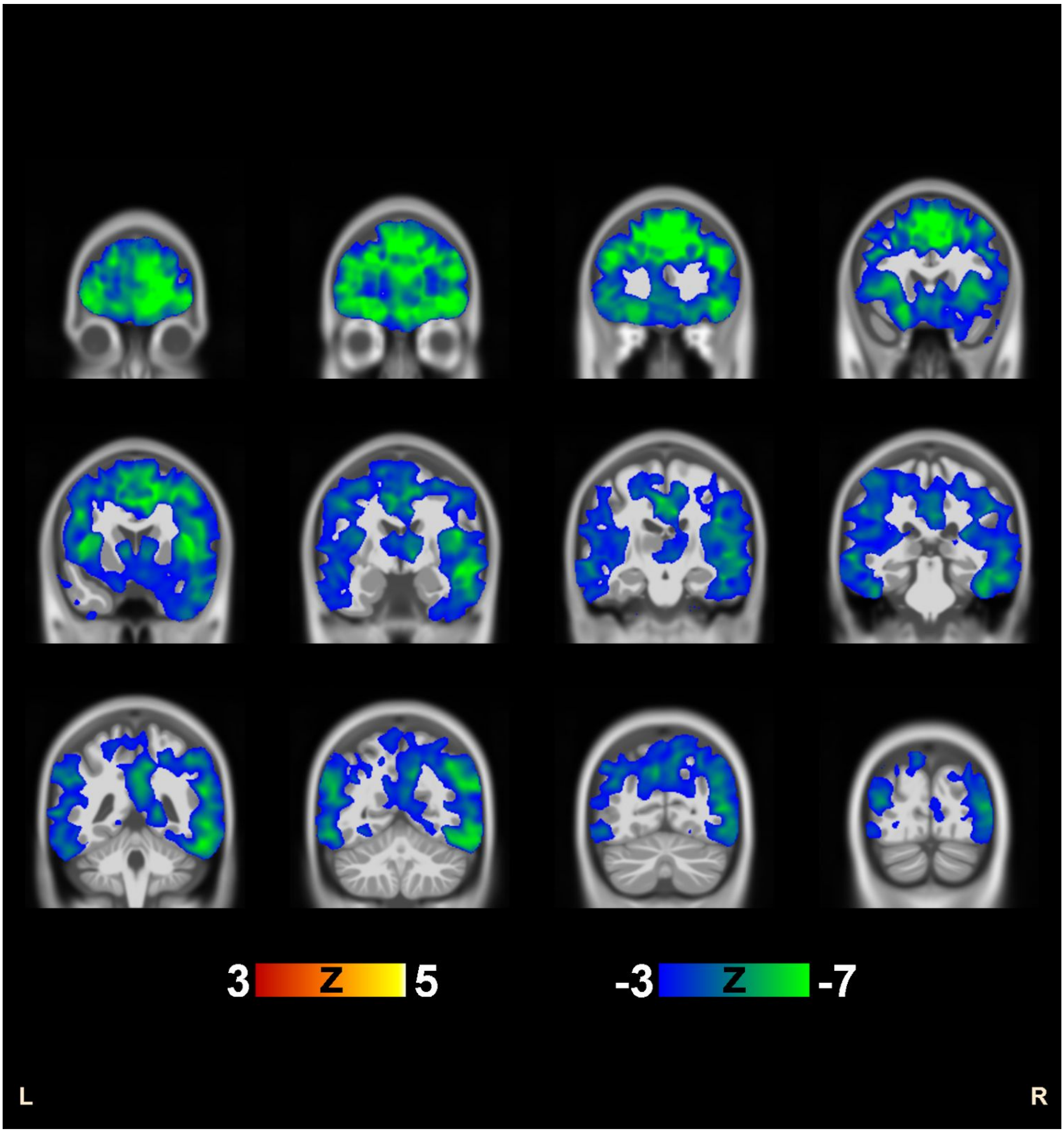

Figure 3 b: Coronal view of the image shown in Figure 3.

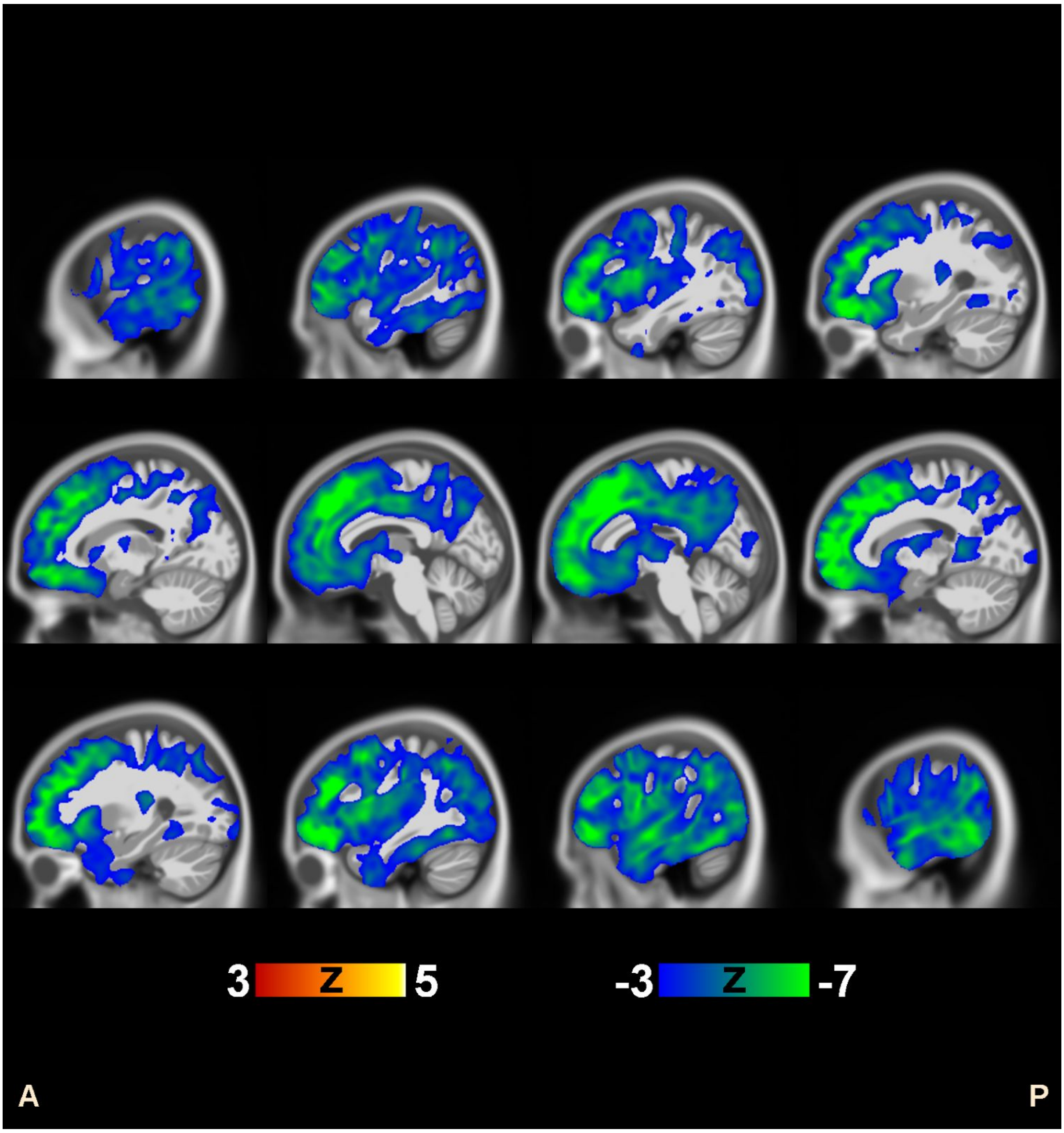

Figure 3 c: Sagittal view of the image shown in Figure 3.

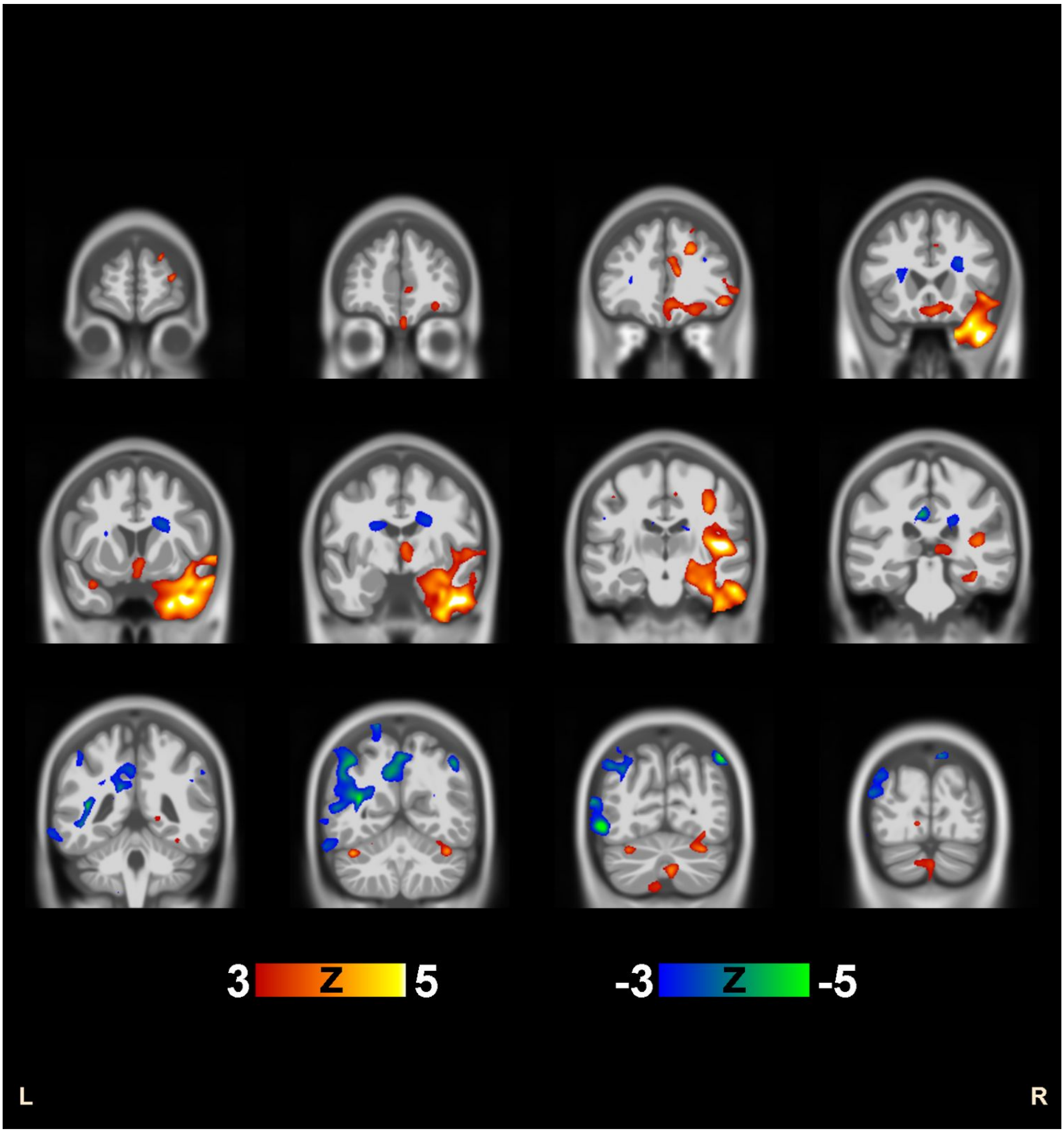

Figure 4 b: Coronal view of the image shown in Figure 4.

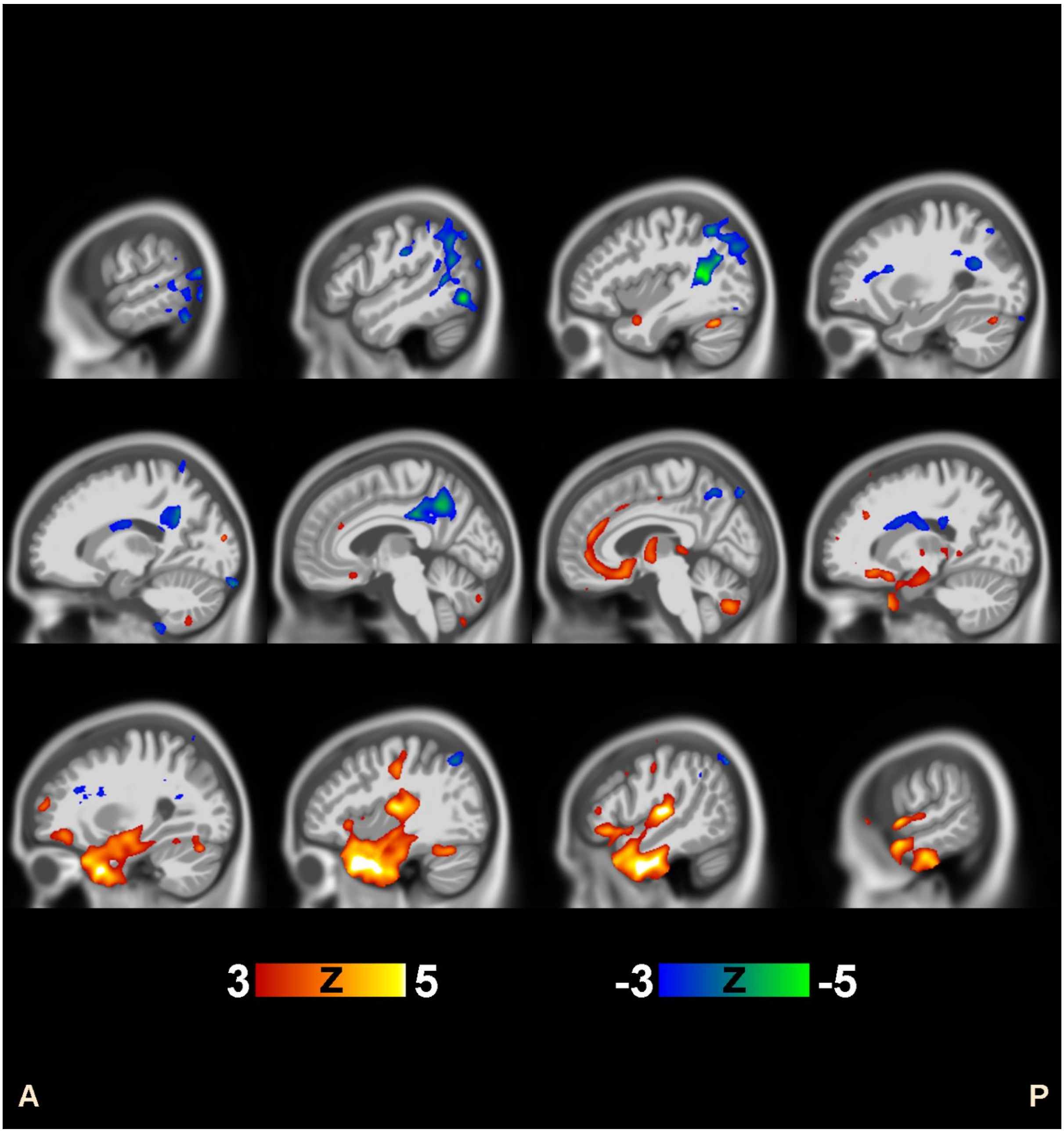

Figure 4 c: Sagittal view of the image shown in Figure 4.

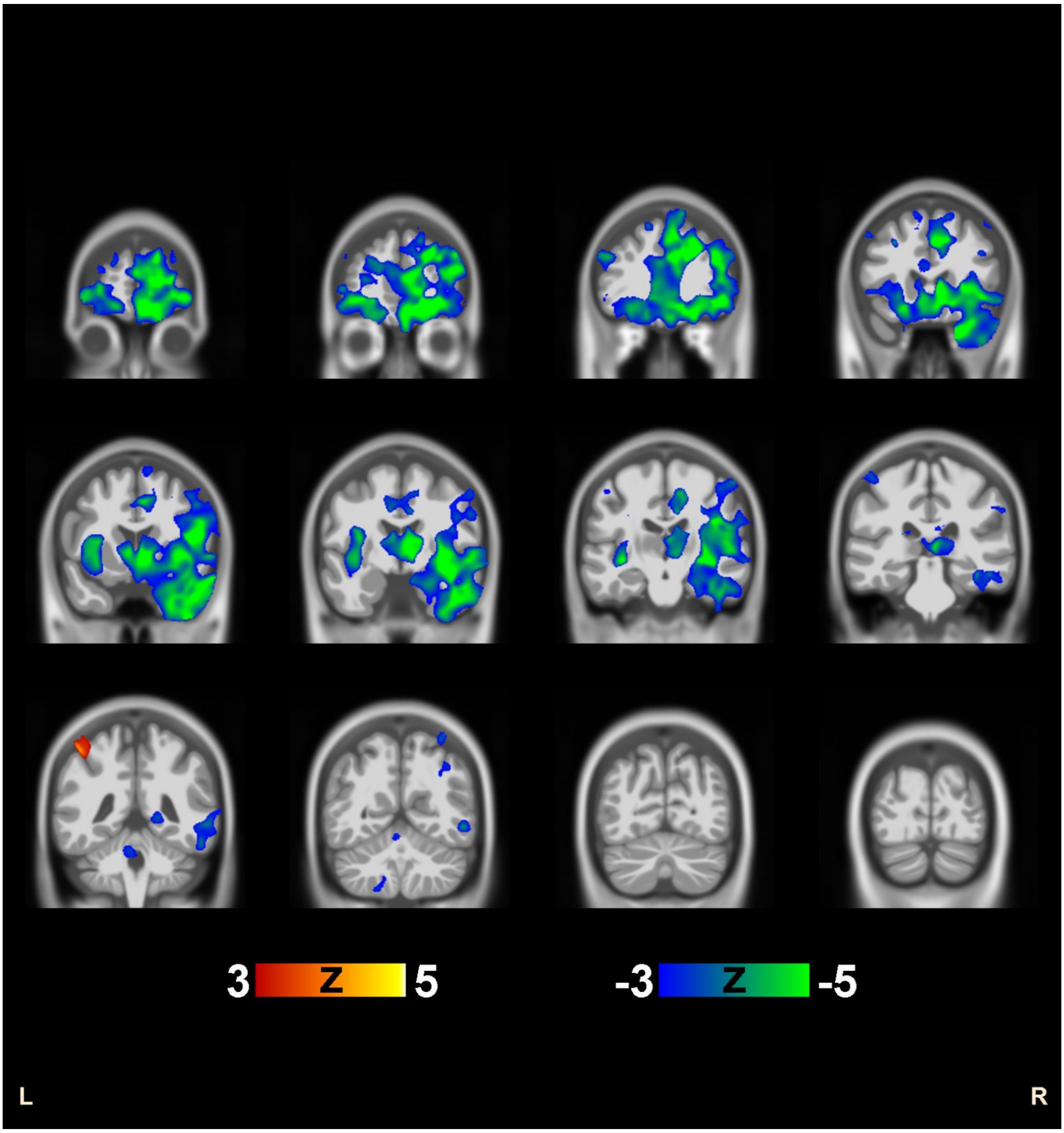

Figure 5 b: Coronal view of the image shown in Figure 5.

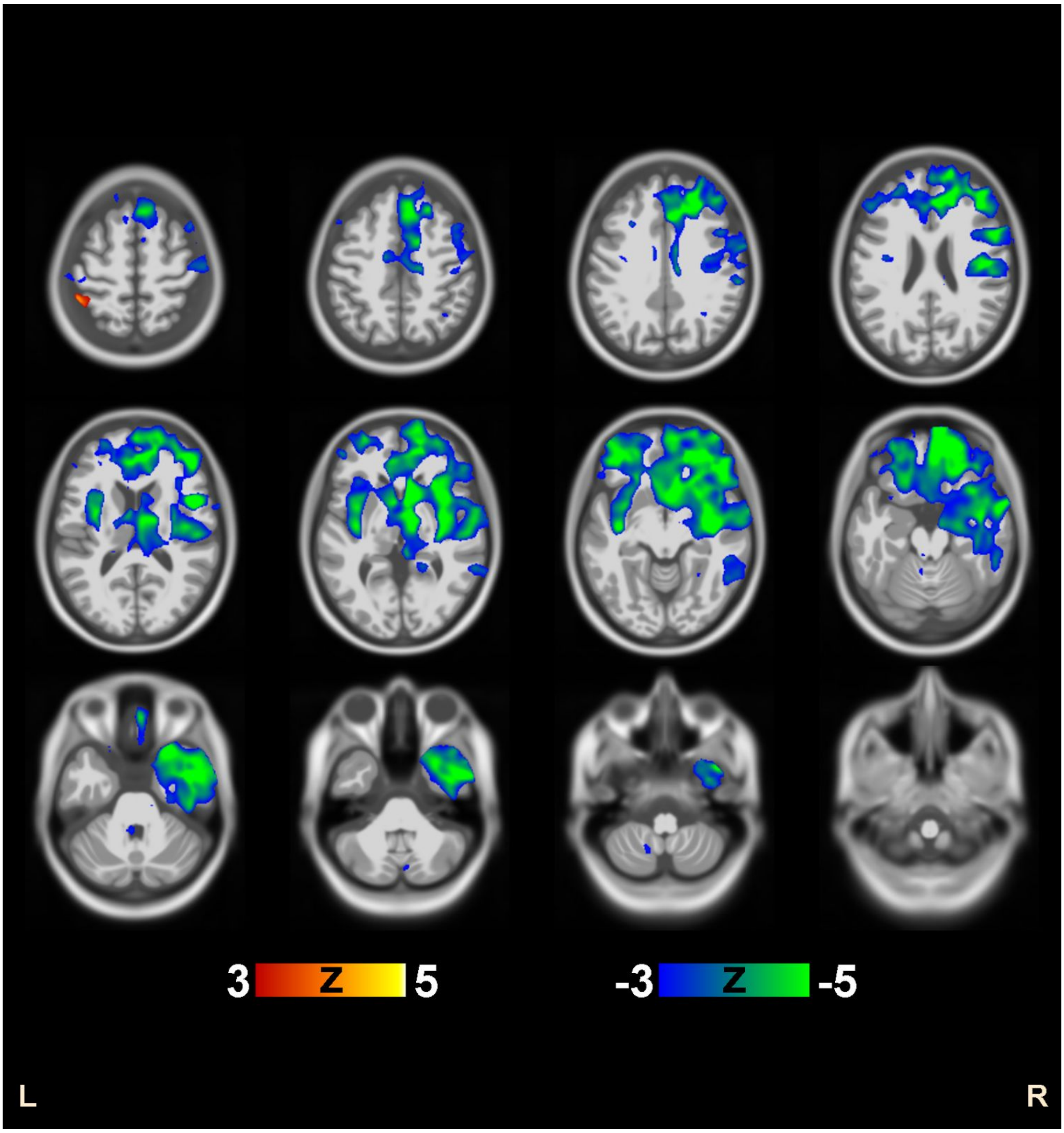

Figure 5 c: Transverse view of the image shown in Figure 5.

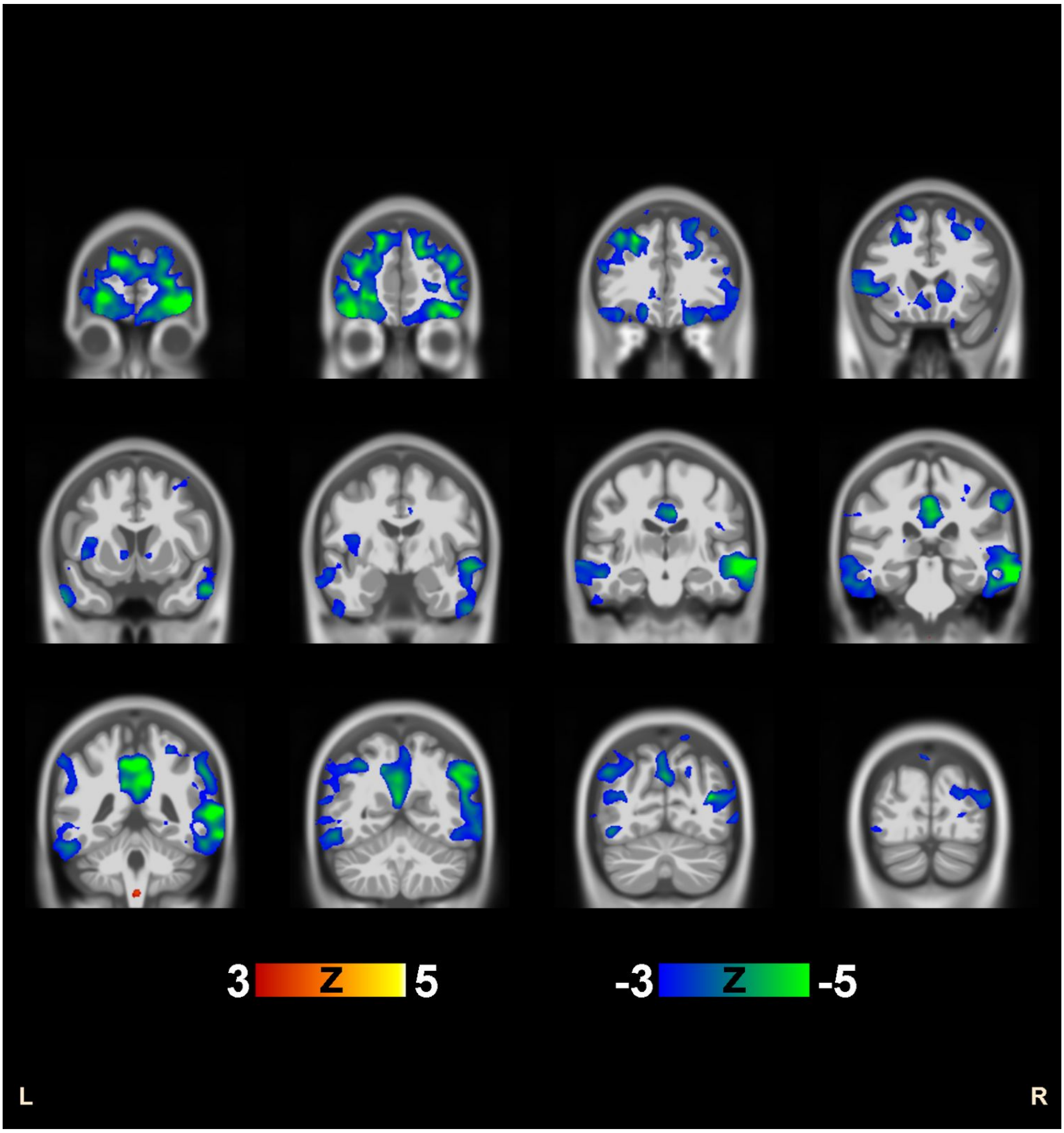

Figure 6 b: Coronal view of the image shown in Figure 6.

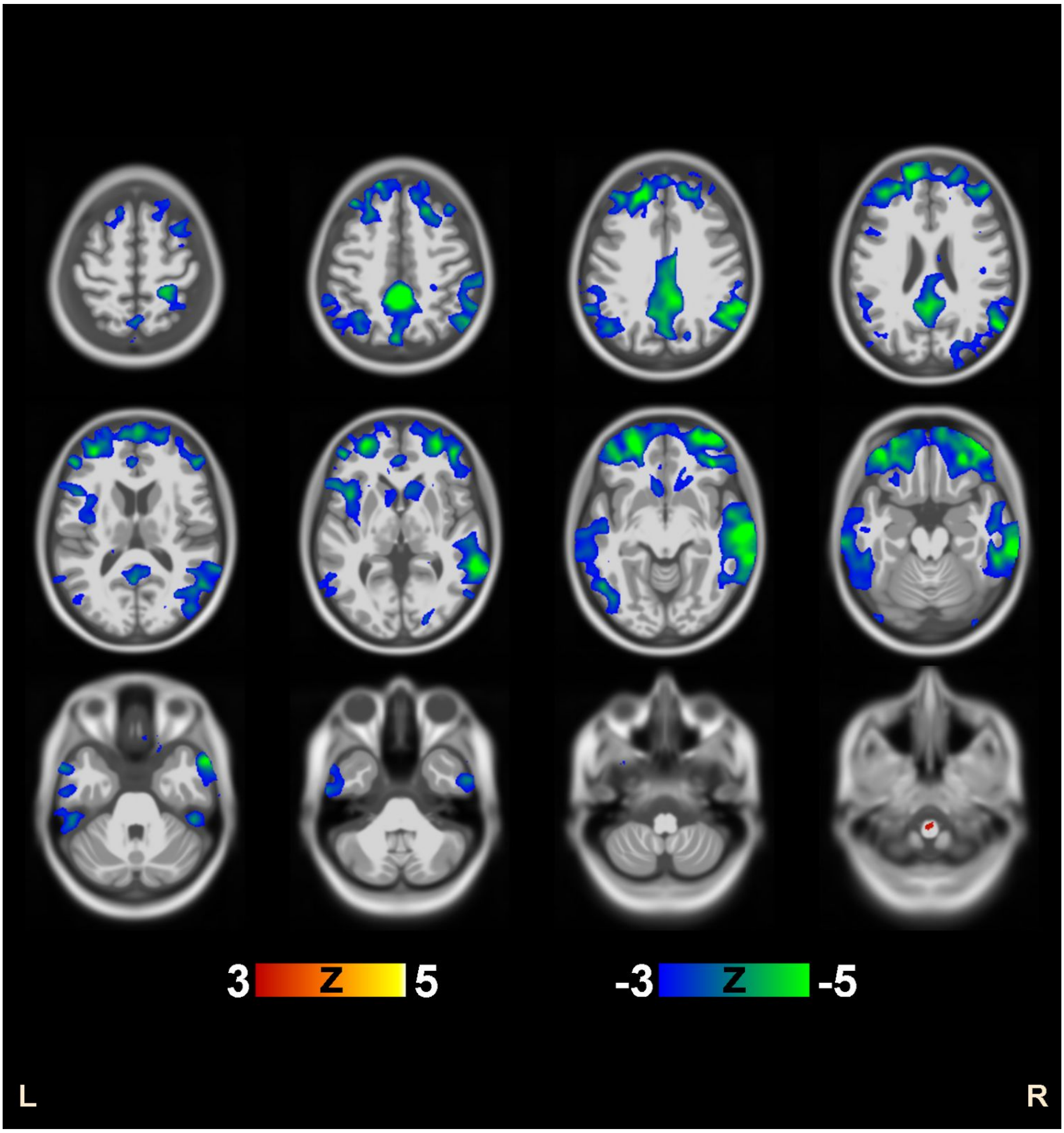

Figure 6 c: Transverse view of the image shown in Figure 6.

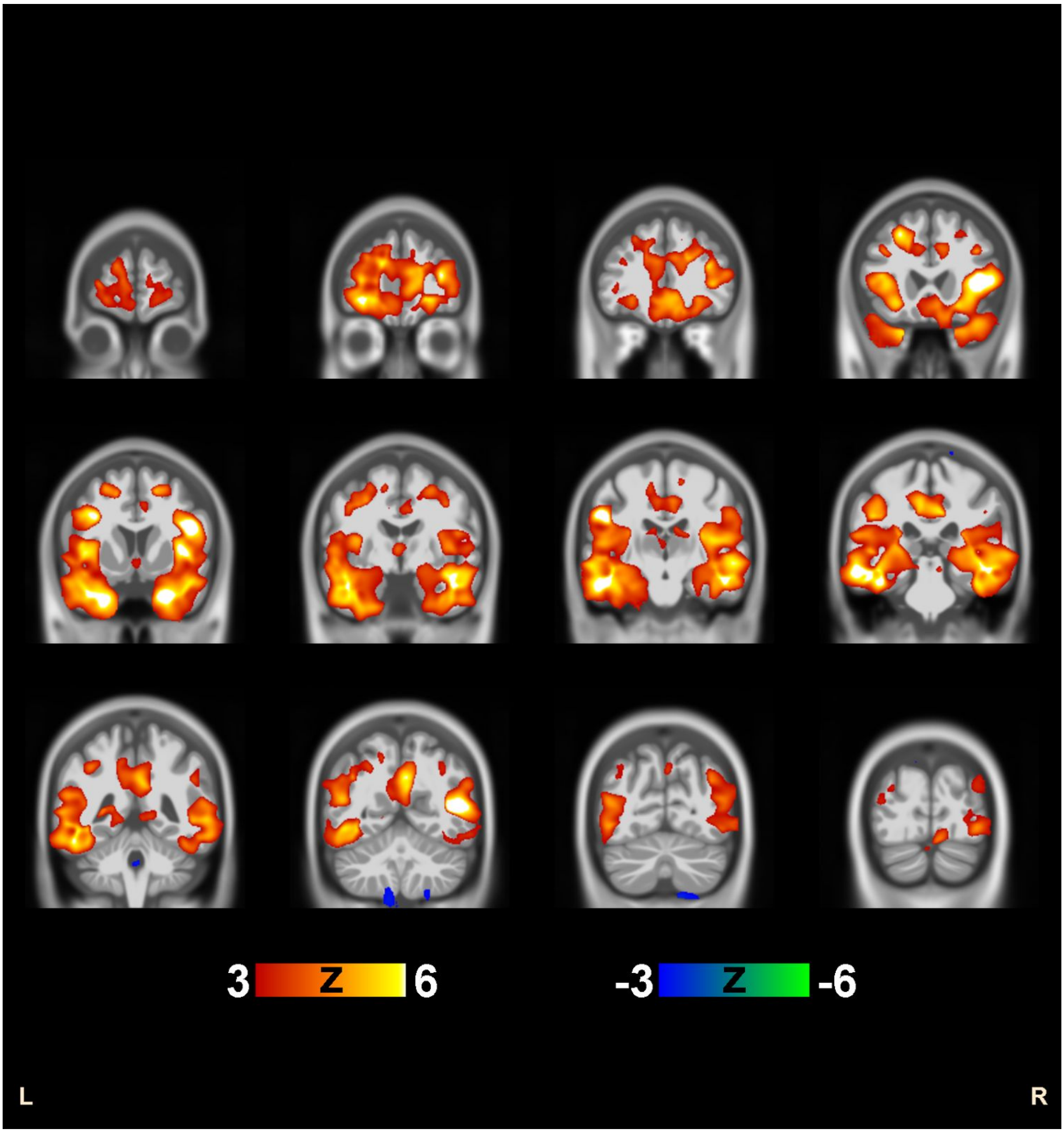

Figure 7 b: Coronal view of the MRIs in Figure 7.

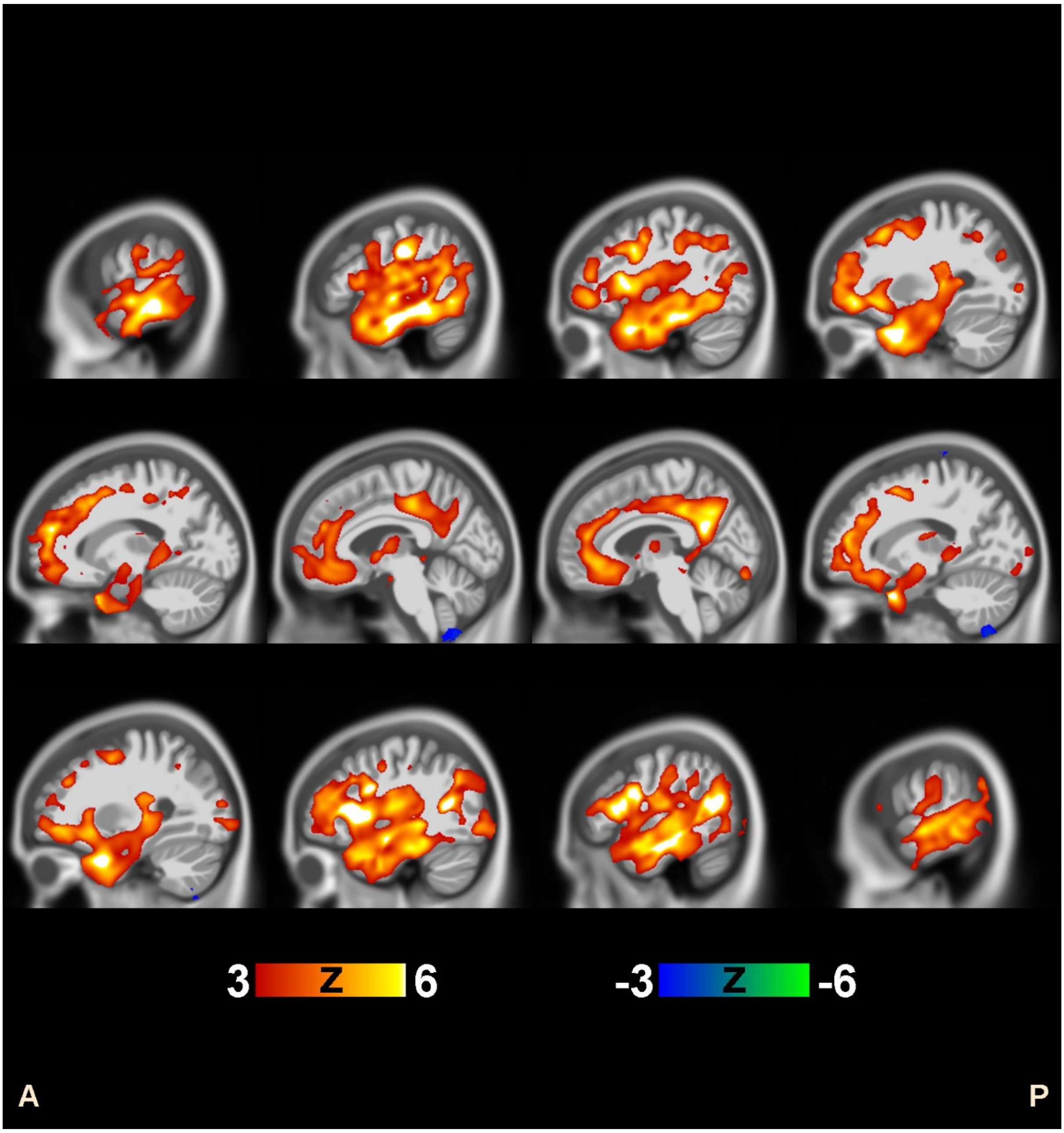

Figure 7 c: Sagittal view of the MRIs in Figure 7.

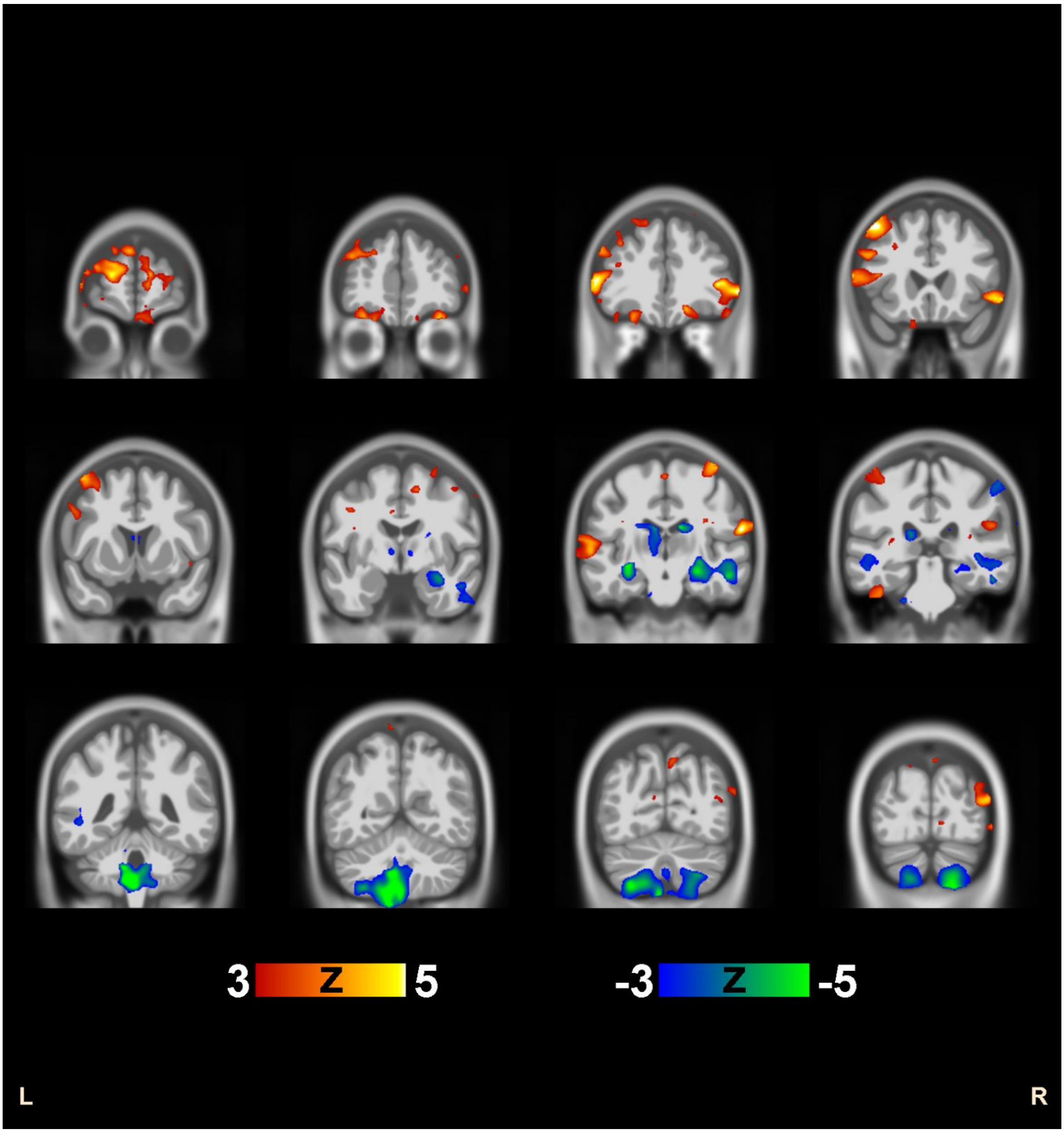

Figure 8 b: Coronal view of the MRIs in Figure 8.

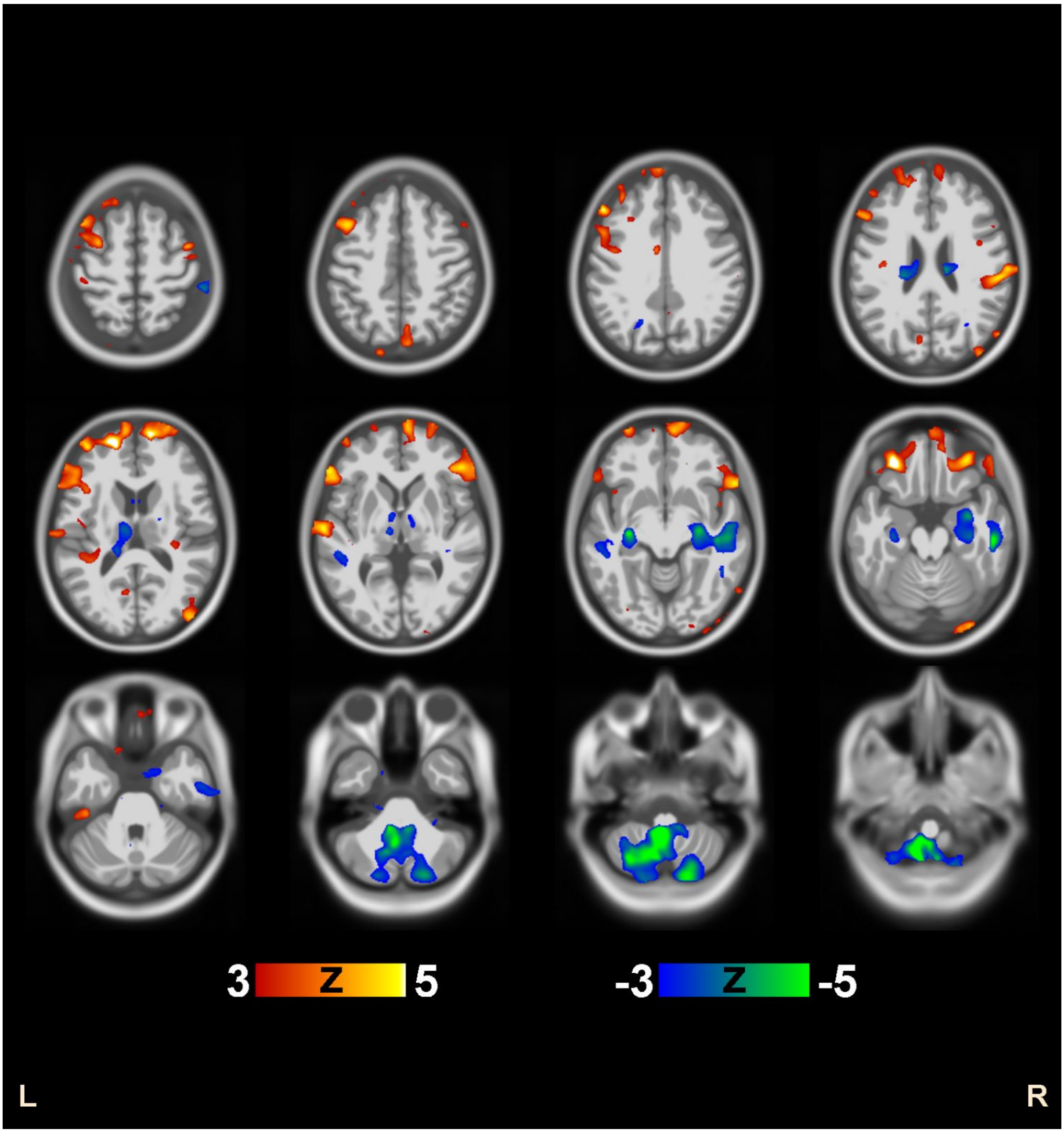

Figure 8 c: Transverse view of the MRIs in Figure 8.

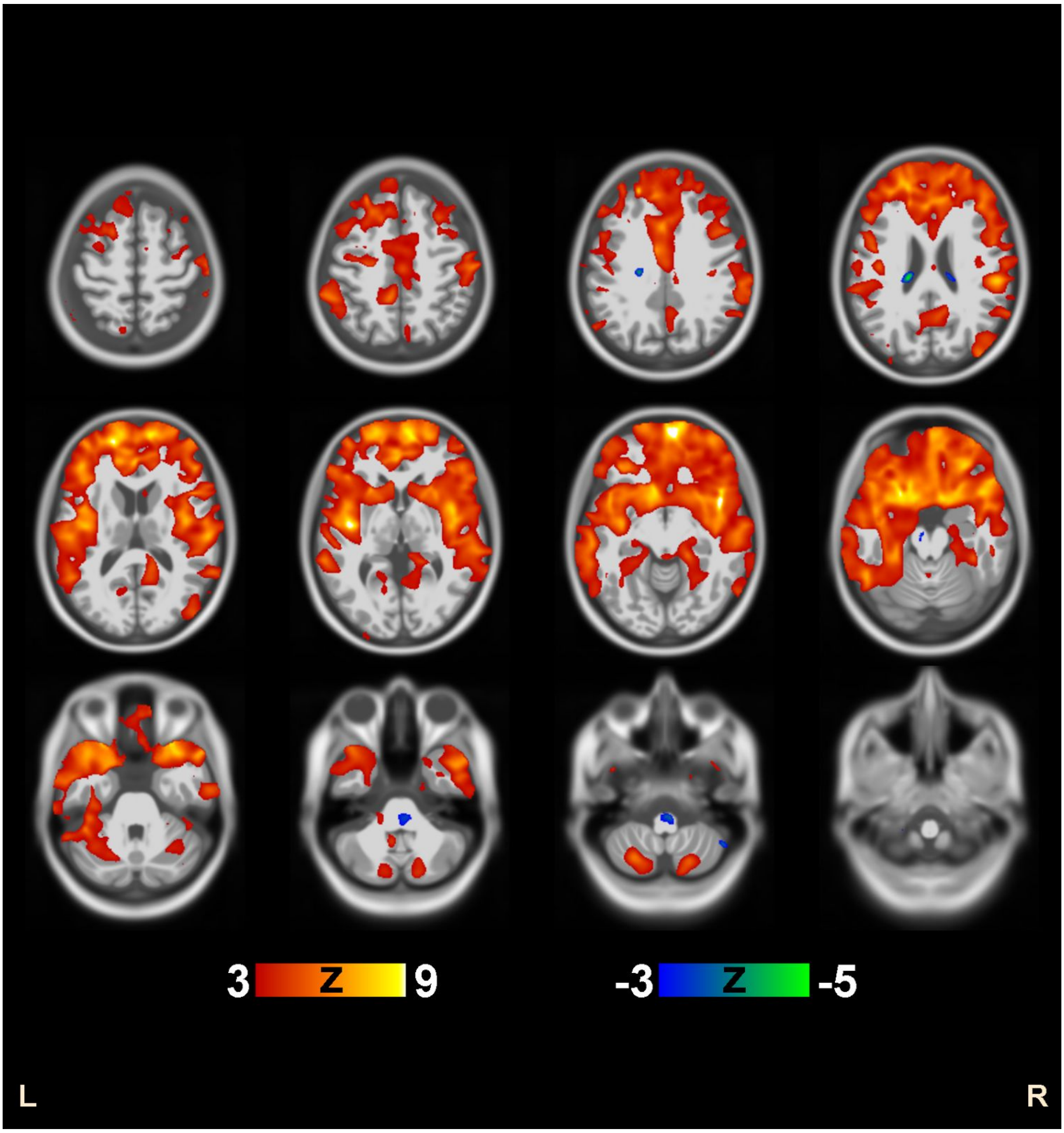

Figure 9 b: Transverse view of the MRIs in Figure 9.

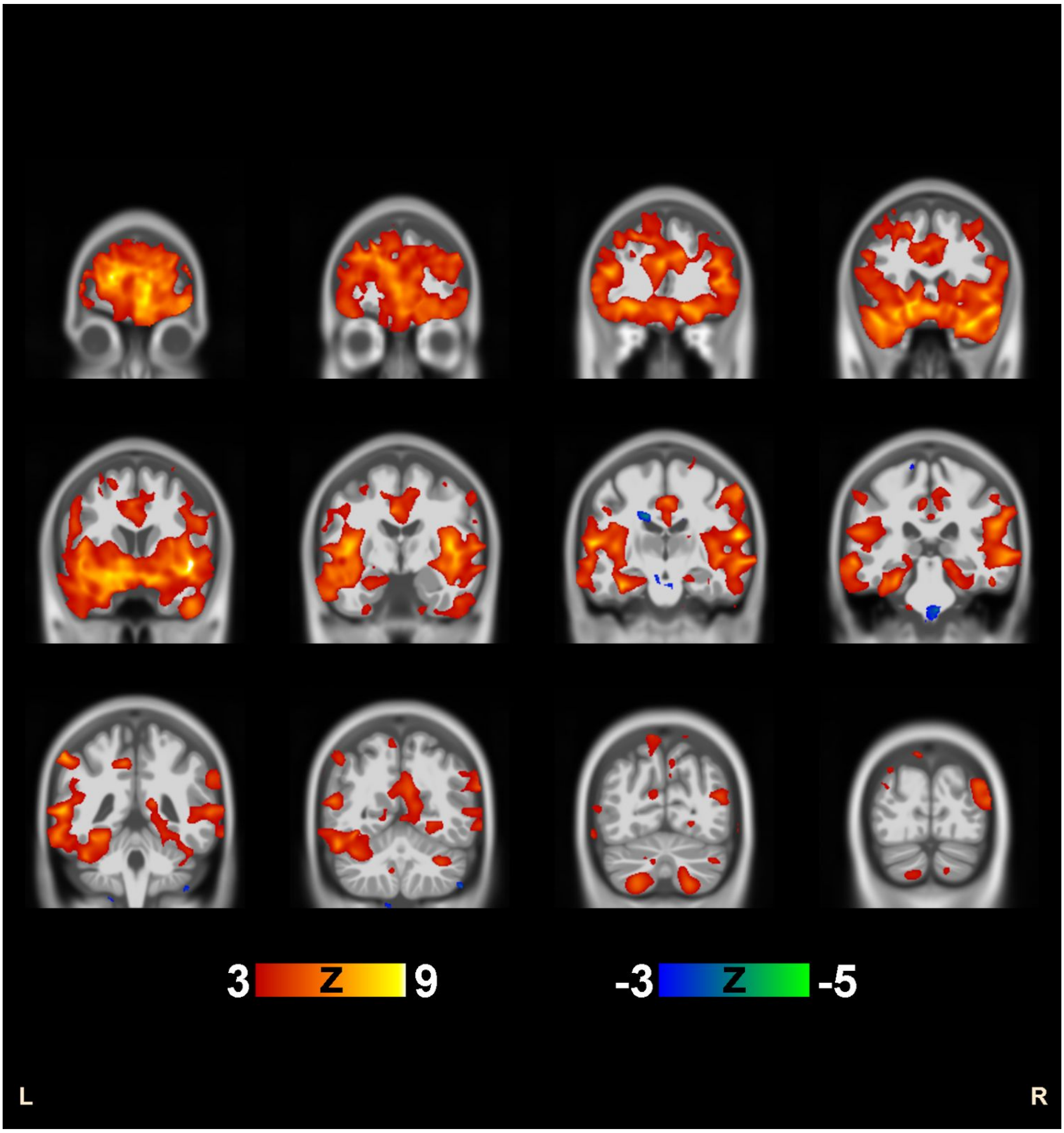

Figure 9 c: Coronal view of the MRIs in Figure 9.

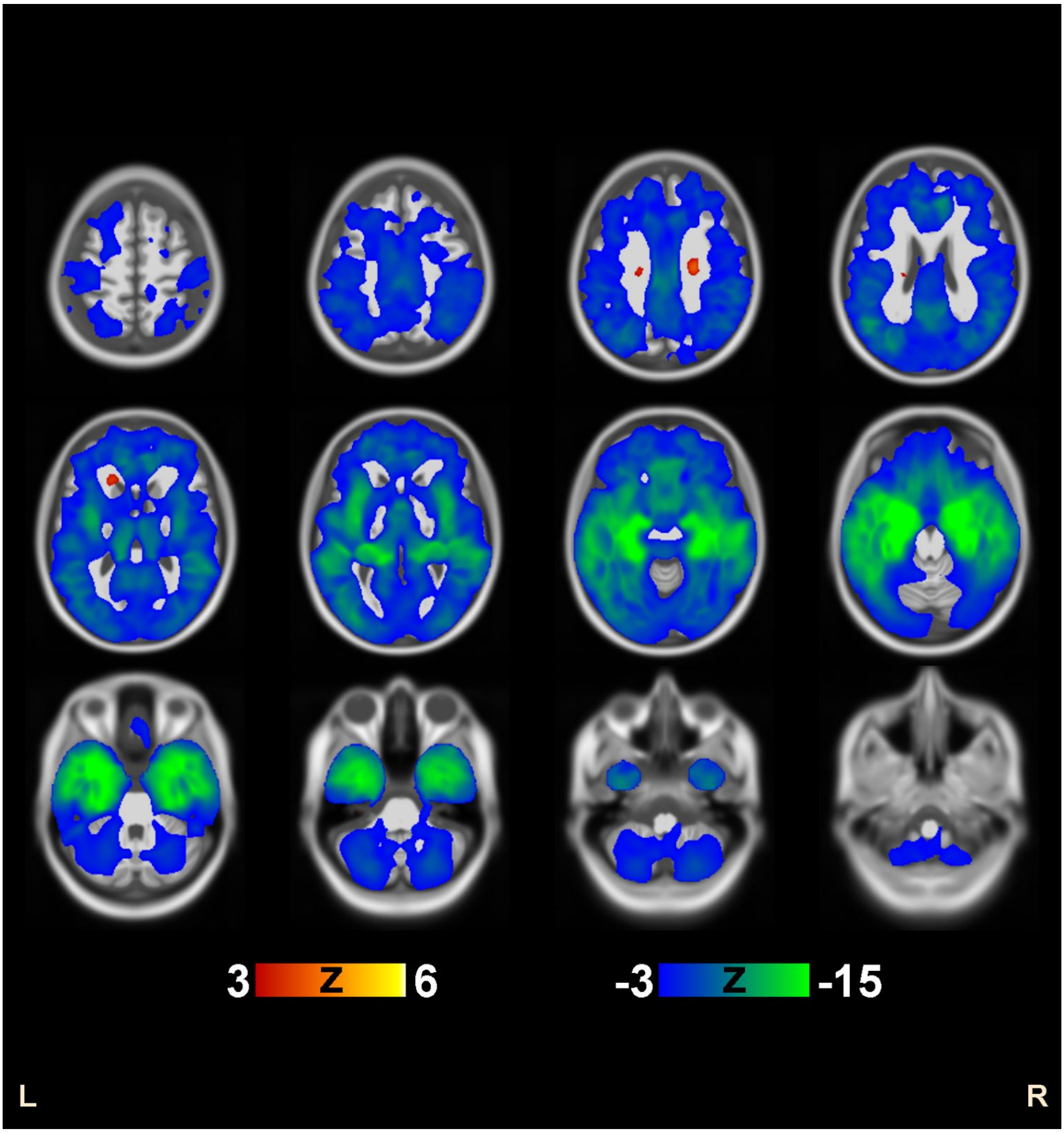

Figure 11: Clusters 1-4 from Table 3 (typical AD) are compared with normal controls (NC). Transverse view, FDR value is 0.000502.

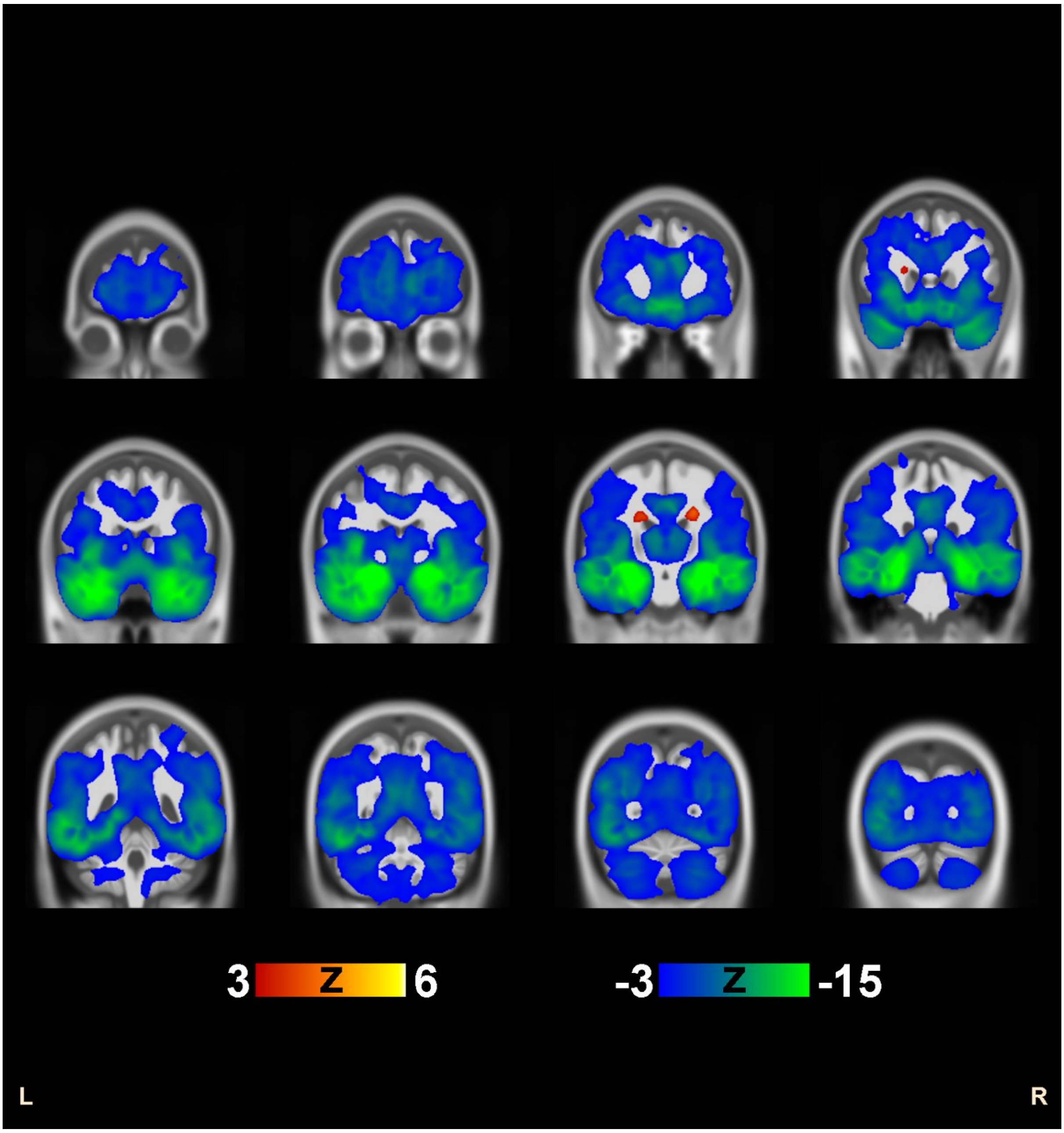

Figure 12: Clusters 1-4 from Table 3 (typical AD) are compared with normal controls (NC). Coronal view, FDR value is 0.000502.

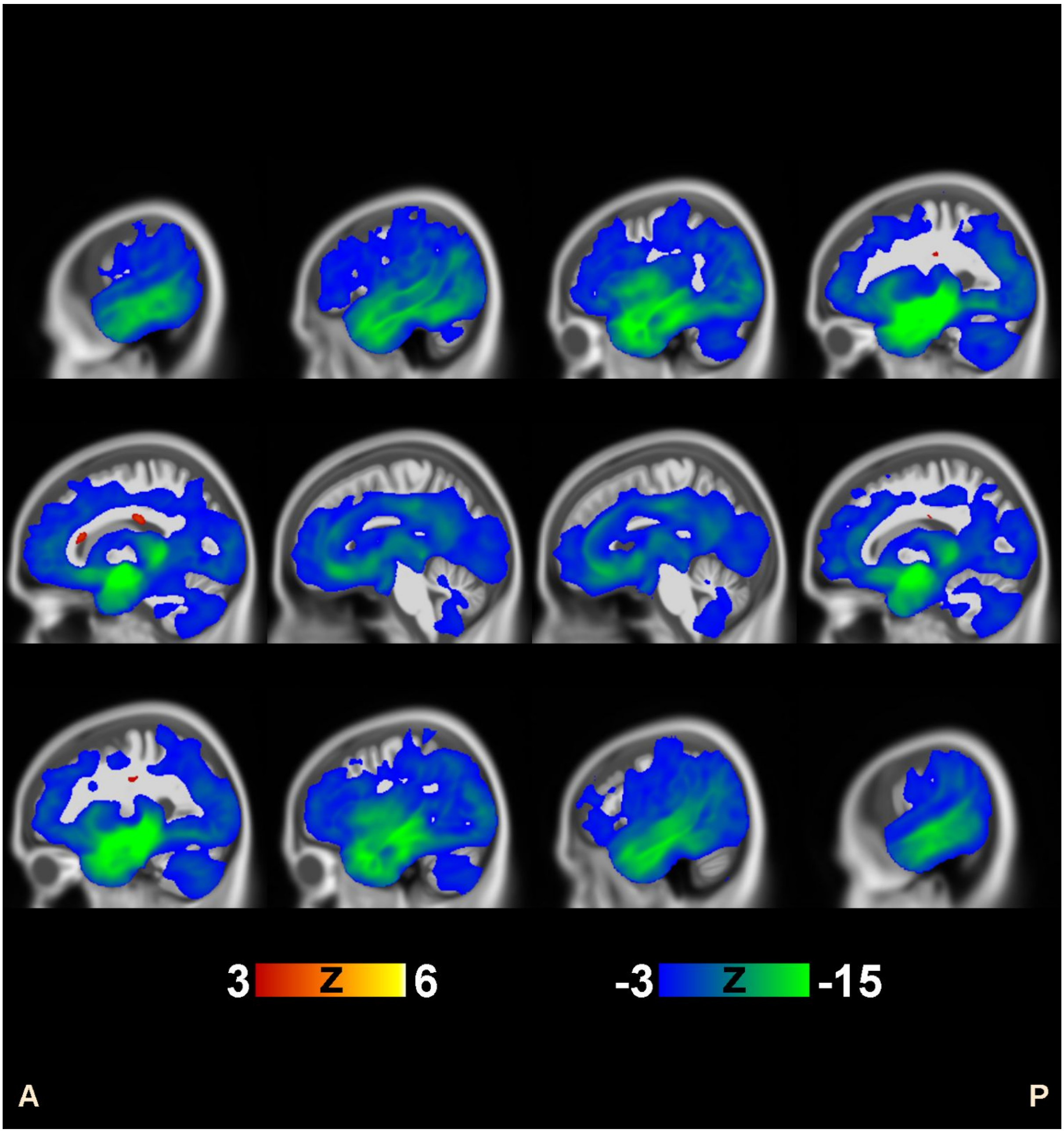

Figure 13: Clusters 1-4 from Table 3 (typical AD) are compared with normal controls (NC). Sagittal view, FDR value is 0.000502.
